# Self-organisation of dodeca-dendronized fullerene into supramolecular discs and helical columns containing a nanowire-like core†
†Electronic supplementary information (ESI) available. See DOI: 10.1039/c5sc00449g



**DOI:** 10.1039/c5sc00449g

**Published:** 2015-04-09

**Authors:** Sebastiano Guerra, Julien Iehl, Michel Holler, Mihai Peterca, Daniela A. Wilson, Benjamin E. Partridge, Shaodong Zhang, Robert Deschenaux, Jean-François Nierengarten, Virgil Percec

**Affiliations:** a Institut de Chimie , Université de Neuchâtel , Avenue de Bellevaux 51 , 2000 Neuchâtel , Switzerland . Email: robert.deschenaux@unine.ch; b Laboratoire de Chimie des Matériaux Moléculaires , Université de Strasbourg et CNRS , Ecole Européenne de Chimie , Polymères et Matériaux , 25 rue Becquerel , 67087 Strasbourg Cedex 2 , France . Email: nierengarten@unistra.fr; c Roy & Diana Vagelos Laboratories , Department of Chemistry , University of Pennsylvania , Philadelphia , Pennsylvania 19104-6323 , USA . Email: percec@sas.upenn.edu

## Abstract

Twelve chiral and achiral self-assembling dendrons have been grafted onto a [60]fullerene hexa-adduct core by copper-catalyzed alkyne azide “click” cycloaddition. The structure adopted by these compounds was determined by the self-assembling peripheral dendrons. These twelve dendrons mediate the self-organisation of the dendronized [60]fullerene into a disc-shaped structure containing the [60]fullerene in the centre. The fullerene-containing discs self-organise into helical supramolecular columns with a fullerene nanowire-like core, forming a 2D columnar hexagonal periodic array. These unprecedented supramolecular structures and their assemblies are expected to provide new developments in chiral complex molecular systems and their application to organic electronics and solar cells.

## Abstract 2

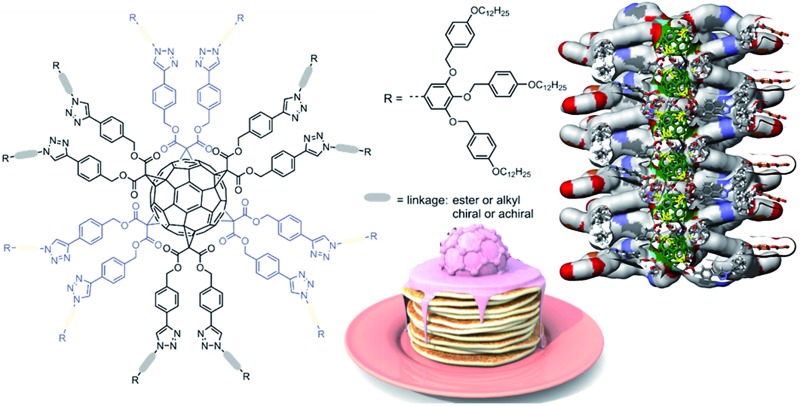
C_60_ dendronized with 12 chiral or achiral self-assembling dendrons form discs with C60 at their centre that self-organise into helical columns with a nanowire-like core.

## 

Primary structure is responsible for the creation of function *via* intramolecular self-assembly to generate a secondary structure, followed by intermolecular self-organisation to generate tertiary and quaternary structures. When this supramolecular principle was applied to the self-assembly of [60]fullerene (C_60_) derivatives in bulk,^1^ novel supramolecular organisations with important applications in organic electronics and photovoltaics were discovered.^2^ Amphiphilic fullerenes self-assemble in water to give a variety of supramolecular structures that have attracted considerable interest for biological applications.^3^ A range of synthetic approaches has provided a large variety of self-assembling C_60_ derivatives with mono-, di-, tetra-, penta-, and hexa-adducts.^4^ A summary of these molecules and of their self-organisation behaviour will be discussed later. Surprisingly, the synthesis and self-organisation of [60]fullerenes functionalized with twelve self-assembling dendrons has not yet been reported.

Here we report the first example of a hexakis-adduct of C_60_ with self-assembling dendrons at every possible position, *i.e.* 12 achiral or chiral dendrons per fullerene. Intuitively, the quasi-spherical hard core of these molecules and an isotropic distribution of dendrons around their surface was expected to force these molecules to adopt a globular shape suitable for self-organisation into various cubic, tetragonal or quasicrystalline periodic and quasiperiodic arrays.^5^ Unexpectedly, regardless of this high degree of substitution of the C_60_ core, the fullerodendrimers reported herein self-organise into 2D columnar arrays, due to the dominating self-assembling ability of the peripheral dendrons.^1e,6^ As in linear dendronized polymers functionalised with related dendrons as side groups,^7^ the 3D structure adopted by the supramolecular system is imposed by the dendritic substituents. The present system represents the largest functional group to be attached to the apex of these self-assembling dendrons to date,^8^ and demonstrates that dendrons of this type maintain their ability to self-organise into columnar arrays,^1e,6^ regardless of the scaffold onto which they were appended.

## Results and discussions

### Brief introduction to self-organising fullerodendrimers

**Fig. 1:**
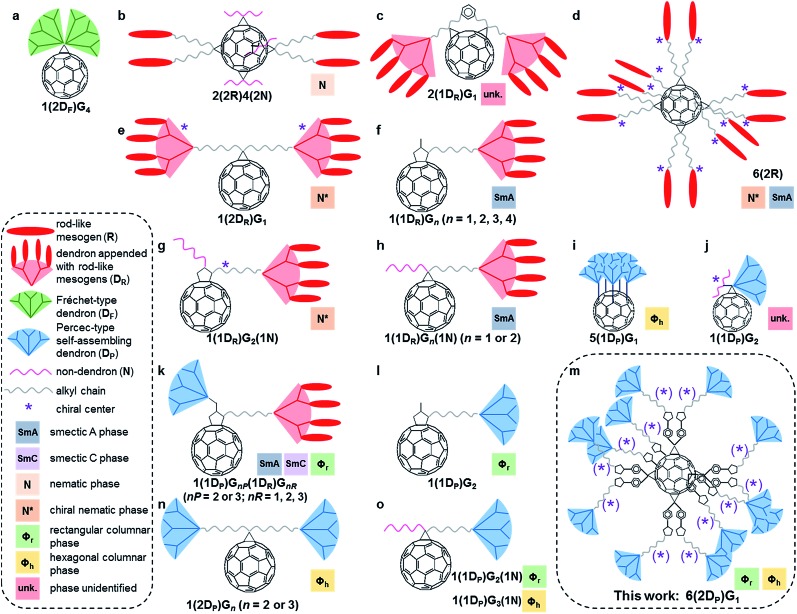
Survey of self-organising fullerodendrimers. The designation *a*(*b*D_X_)G_*n*_ indicates a fullerene substituted in *a* positions with *b* dendrons of type X and generation *n*, where X = F for Fréchet-type dendrons, P for Percec-type self-assembling dendrons, R for rod-like mesogen-appended dendrons and N for non-dendrons.

Dendronized fullerenes are an attractive synthetic target for fundamental studies and practical applications.^1*e*^ Numerous dendronized fullerenes, many of them generating 1D and 2D periodic arrays, have been reported since the first successful synthesis by the Fréchet laboratory of fullerodendrimers in 1993 (Fig. 1a).^1,4^ Fréchet's mono-adduct was dendronized with two fourth-generation Fréchet-type dendrons.^9^ Subsequent efforts to generate self-organising fullerodendrimers utilized rod-like mesogenic groups (Fig. 1b–h),^4a–g^ which induced the formation of smectic A, nematic and chiral nematic phases. The mesogenic rod-like molecules were either appended to first-, second-, third- or fourth-generation non-self-assembling aryl ester dendrons (Fig. 1c and e–h) or attached directly to the bridge of methanofullerenes (Fig. 1b and d). In the latter case, the sole dendritic branching point in the molecule is the C_60_ core, and the resultant compounds are best classified as first-generation dendrimers rather than as a fullerene appended with dendrons.

Self-assembling dendrons have been attached to fullerene by several different laboratories, allowing the formation of previously inaccessible columnar assemblies by fullerodendrimers. The ‘shuttlecock’ fullerodendrimer reported by the Kato and Nakamura laboratories (Fig. 1i) formed columnar hexagonal 2D phases *via* the stacking of the fullerene moiety within a ‘cup’ defined by dendrons.^4*h*^ More recent work has utilized self-assembling dendrons of the second generation or higher, functionalized with polypeptides to form filaments (Fig. 1j),^4*i*^ with rod-like mesogens that form Janus-type fullerodendrimers exhibiting smectic and columnar phases (Fig. 1k),^4*j*^ or without further substitution to yield exclusively columnar periodic arrays (Fig. 1l, n and o).^4k,l^ Aside from Kato and Nakamura's ‘shuttlecock’, there have been no reports of fullerenes with more than two self-assembling dendrons, nor any utilizing only first generation self-assembling dendrons to achieve columnar self-organisation. Thus the molecules reported here and outlined in Fig. 1m represent the most highly substituted dendronized fullerenes capable of self-organisation to date.

### Synthesis of dendronized fullerene

**Scheme 1:**
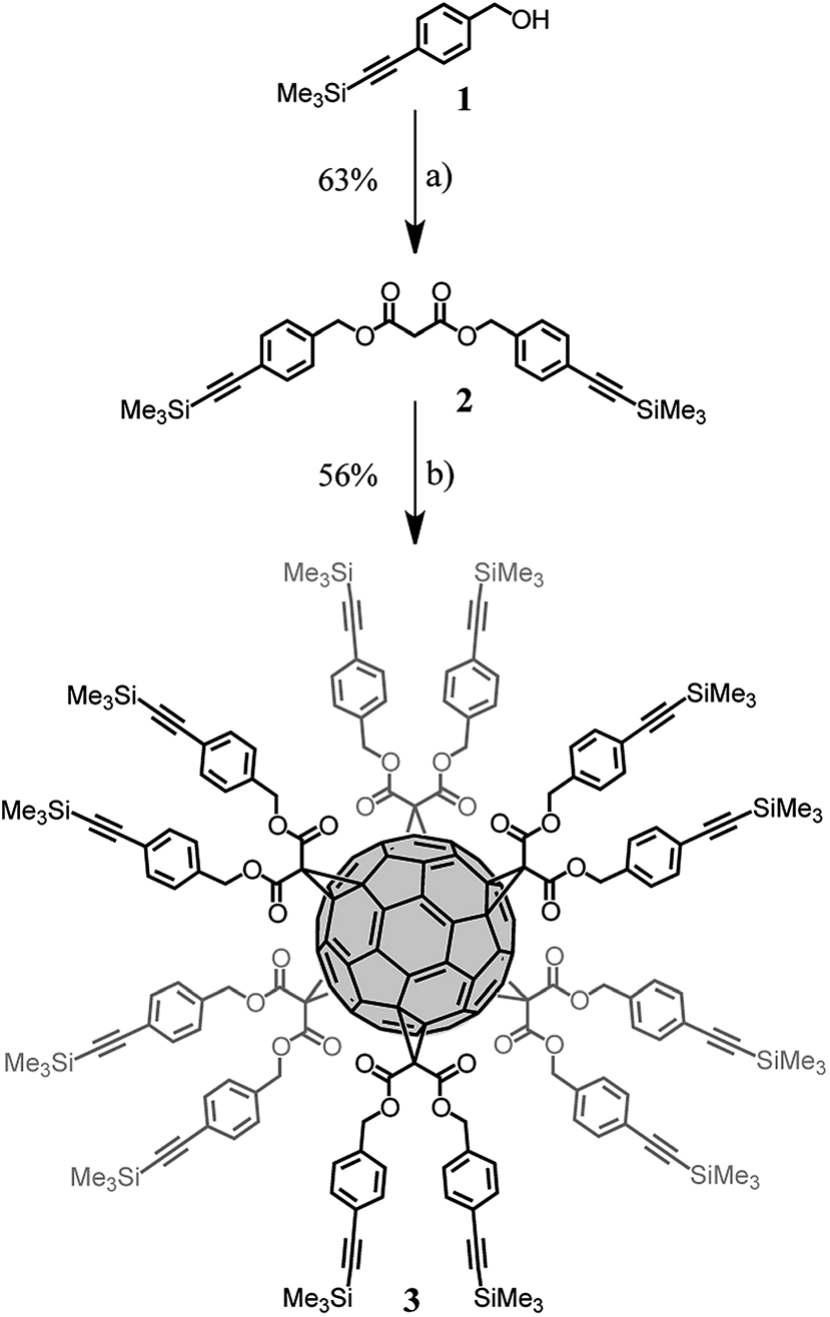
Synthesis of compound **3**. Reagents and conditions: (a) malonyl chloride, pyridine, CH_2_Cl_2_, 0 °C to RT, 16 h (63%); (b) C_60_, DBU, CBr_4_, *o*-DCB, RT, 72 h (56%).

Fullerene hexakis-adducts with a *T*_h_-symmetrical addition pattern^1*a*^ were prepared in a single step by direct treatment of C_60_ with malonates. These reaction conditions, developed by Hirsch^10^ and refined by Sun,^11^ are very sensitive to steric factors.^11^ The reaction of C_60_ with malonates bearing large substituents provided the hexa-adducts in low yields and their purification was often difficult. Therefore, we selected a synthetic route based on the post-functionalization of a pre-constructed fullerene hexa-adduct derivative with dendrons having complementary functionality.^12^ For this purpose, copper-catalysed alkyne azide 1,3-dipolar cycloaddition (CuAAC)^13^ was selected to perform the conjugation, as this reaction has been successfully applied to the efficient synthesis of a large diversity of sophisticated fullerene hexa-adducts.^14^ The preparation of the key fullerene building block **3** is shown in Scheme 1. Treatment of **1** with malonyl chloride in the presence of pyridine gave malonate **2** in 63% yield. Fullerene hexa-adduct **3** was obtained by the reaction of malonate **2** with C_60_ under the conditions developed by Sun.^11^ Specifically, treatment of C_60_ (1 equiv.) with **2** (10 equiv.), CBr_4_ (100 equiv.) and 1,8-diazabicyclo[5.4.0]undec-7-ene (DBU, 20 equiv.) in *o*-dichlorobenzene (*o*-DCB) at room temperature for 72 h gave **3** in 56% yield. The detailed structural analysis of this compound by ^1^H and ^13^C NMR and MALDI-TOF is available in the ESI.†

**Scheme 2:**
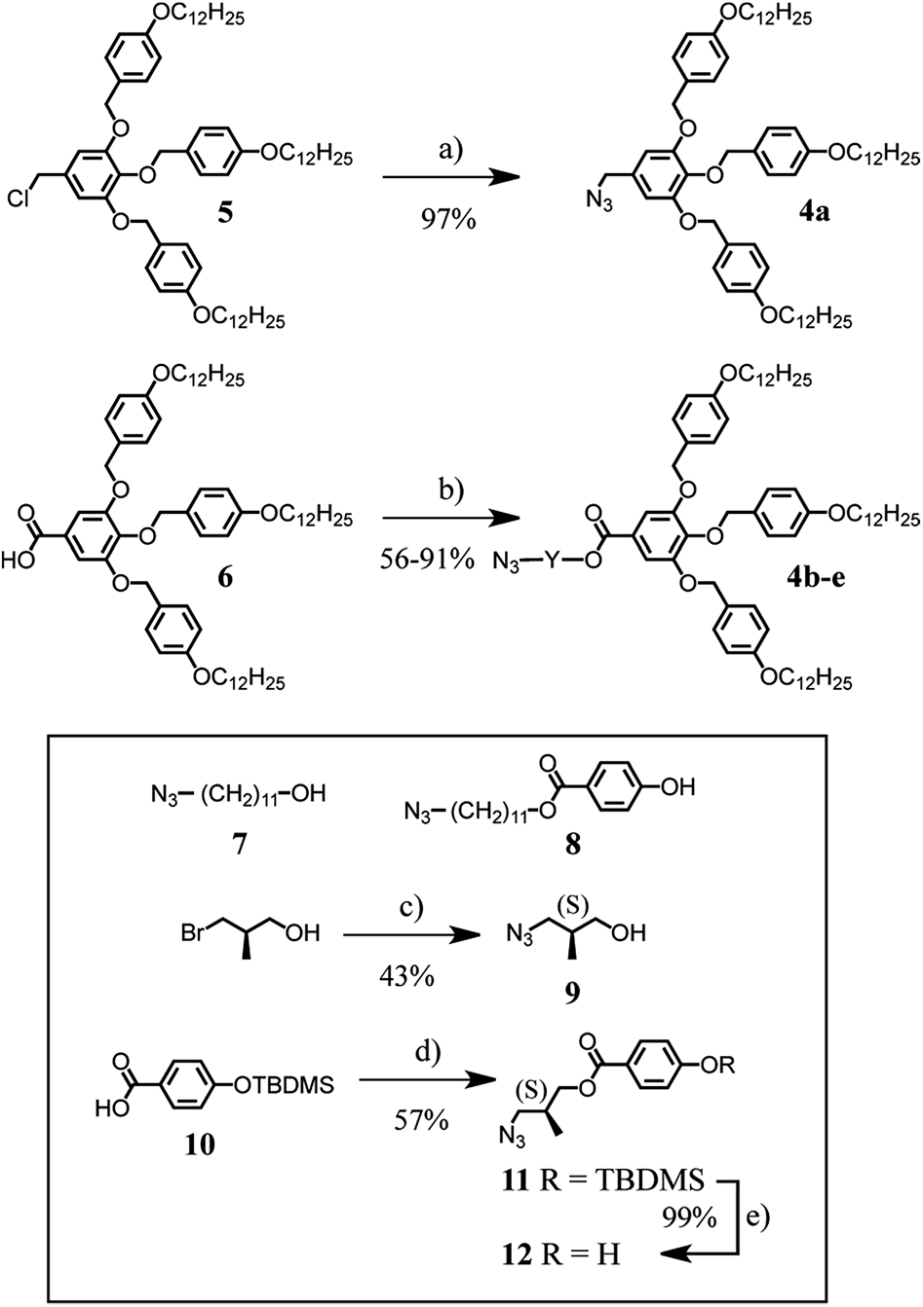
Synthesis of dendrons **4a–e**. Reagents and conditions: (a) NaN_3_, DMF, 70 °C, 24 h (97%); (b) HO–Y–N_3_ (**7**, **8**, **9** or **12**), DPTS, EDC, CH_2_Cl_2_, 0 °C to RT, 24 h [from **7**: **4b** (56%), from **8**: **4c** (86%), from **9**: **4d** (91%), from **12**: **4e** (71%),]. Inset: structure of compounds **7**–**8** and preparation of **9** and **12**. Reagents and conditions: (c) NaN_3_, DMF, 70 °C, 24 h (43%); (d) **9**, DPTS, EDC, CH_2_Cl_2_, 0 °C to RT, 24 h (57%); (e) ZnBF_4_·6–7H_2_O, THF/H_2_O, 50 °C, 24 h (99%).

The synthesis of the first generation self-assembling dendron^1e,15^ with azide at the focal point, is depicted in Scheme 2. The difference between these building blocks is the length of the spacer between the dendritic part and the azide group. **4a** has only one CH_2_ spacer while the others incorporate either a (CH_2_)_11_ spacer, **4b**, or a (CH_2_)_11_ spacer with an additional aromatic ring, **4c**. Compounds **4d**, **4e** contain an optically pure spacer derived from *R*-(–)-3-bromo-2-methyl-1-propanol. Structures **5** and **6** were prepared according to literature procedures.^6^ Compound **4a** was obtained in 97% yield by reaction of chloride **5** with NaN_3_ in DMF at 70 °C. Reaction of the carboxylic acid **6** with alcohol **7** under esterification conditions using *N*-(3-dimethylaminopropyl)-*N*′-ethylcarbodiimide (EDC) and 4-(dimethylamino)pyridinium 4-toluenesulfonate (DPTS) led to dendron **4b** in 56% yield. EDC-mediated esterification of **6** with alcohol **8** afforded **4c** in 86% yield. Treatment of *R*-(–)-3-bromo-2-methyl-1-propanol with NaN_3_ in DMF gave **9** and subsequent esterification with **6** afforded dendron **4d**. The second optically active dendron (**4e**) was obtained in 71% yield under similar conditions from carboxylic acid **6** and phenol **12**. This compound was obtained by EDC-mediated esterification of **10** with alcohol **9** followed by desilylation with ZnBF_4_. Analytical data for all compounds are in ESI.†

**Scheme 3:**
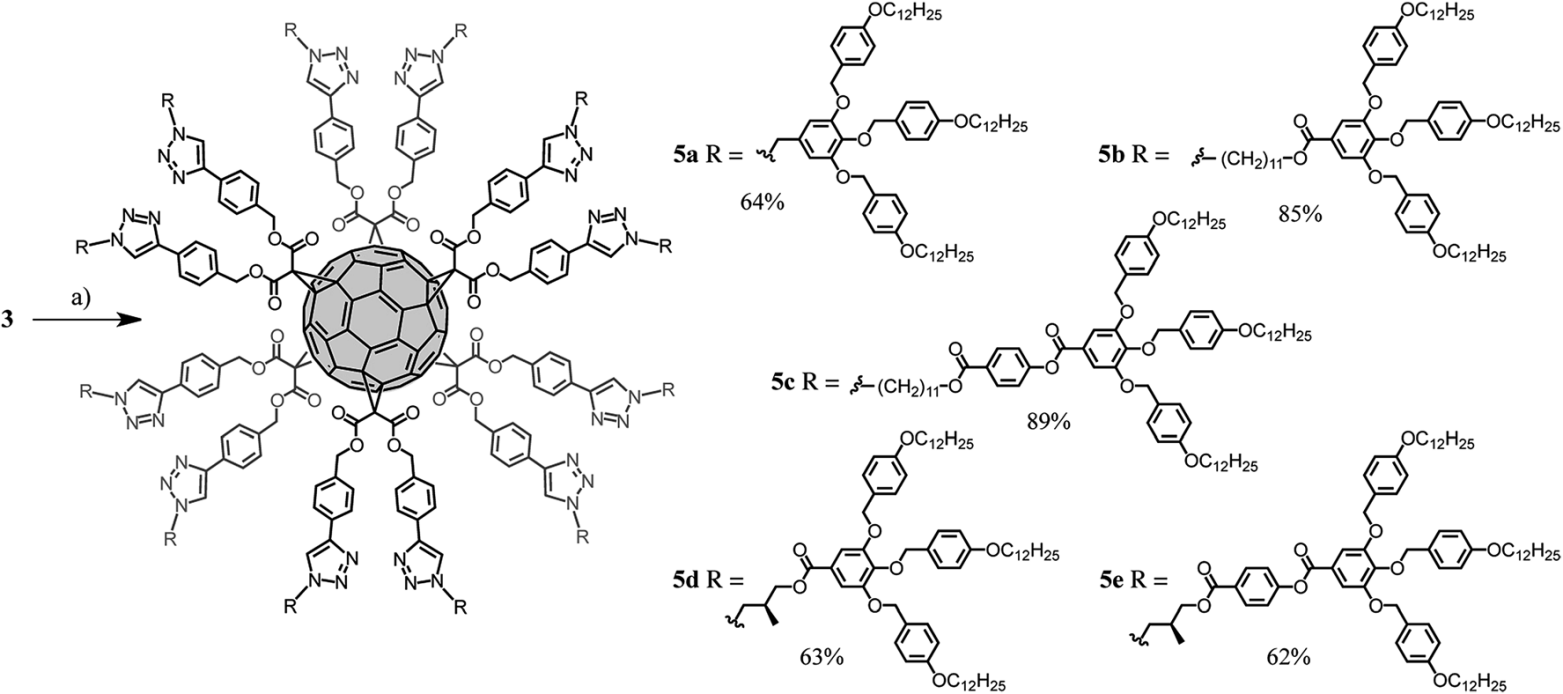
Synthesis of compounds **5a–e**. Reagents and conditions: (a) **4a–e**, TBAF, CuSO_4_·5H_2_O, sodium ascorbate, CH_2_Cl_2_·H_2_O (**5a**: 64%, **5b**: 85%, **5c**: 89%, **5d**: 63%, **5e**: 62%).

The synthesis of the dendronized fullerene **5a–e** is shown in Scheme 3. Compound **3** was desilylated by treatment with an excess of tetrabutylammonium fluoride (TBAF) to provide the corresponding fullerene hexa-adduct bearing the 12 terminal alkyne units requested for its subsequent functionalization under CuAAC conditions. However, the yield of this deprotection was not reproducible and partial decomposition was sometimes observed. Therefore it was more convenient to perform the desilylation *in situ* during the CuAAC reaction.^16^ Indeed, treatment of compound **3** with azides **4a–e** in the presence of a desilylating agent (TBAF), CuSO_4_·5H_2_O and sodium ascorbate provided compounds **5a–e** in good yield (62–89%). The structures of all dendronized fullerenes were confirmed by a combination of IR, UV-vis, ^1^H and ^13^C NMR, and elementary analysis. These analytical data are available in ESI.† The structure of compounds **5b** and **5c** was also confirmed by MALDI-TOF mass spectrometry, showing the expected molecular ion peaks (Fig. S16†). Under the same experimental conditions, the molecular ion peak could not be detected for the molecules incorporating the shortest spacer (**5a**, **5d** and **5e**). This is not the result of high levels of fragmentation, as characteristic fragments were also not observed, but may be related to aggregation effects preventing the transfer of the compounds or fragments thereof in the gas phase during MALDI-TOF analysis. The ^1^H and ^13^C NMR spectra recorded for compounds **5a–e** are in perfect agreement with their *T*_h_ symmetrical structures, and their UV-vis spectra revealed characteristic absorption features of fullerene hexa-adducts (Fig. S1–S16†).^17^

### Structural analysis of supramolecular assemblies

The self-organisation of derivatives **4a–e** and dendronized fullerene hexakis-adducts **5a–e** has been investigated by a combination of techniques including differential scanning calorimetry (DSC), polarized optical microscopy (POM), X-ray diffraction (XRD) on powder and oriented fibre specimens together with electron density maps, and circular dichroism (CD). The phase transition temperatures and the corresponding enthalpies are summarized in Table 1.

**Table 1:** Transition temperatures^*a*^ and associated enthalpies of compounds **4a–c** and **5a–c**

Compound	Thermal transition (°C) and corresponding enthalpy changes (kcal mol^–1^)
**4a**	k 34(15.80) Φ_r-s_ 60(41.63) i
**4b**	k 30(19.00) i
**4c**	k 33(28.94) i
**4d**	k 48(26.41) i
**4e**	k 33(25.17) i
**5a**	Φ_h_ 160(3.32) i
**5b**	Φ_h_ 114(3.20) i
**5c**	Φ_h_ 137(1.67) Φ_r-c_ 154(5.95) i
**5d**	Φ_h_ *ca.*110 i
**5e**	Φ_h_ 131(0.96) i

^*a*^Transition temperatures, their associated enthalpy changes and assignments were determined from second heating scans by DSC with 10 °C min^–1^ and XRD. Phase notation: Φ_r-s_ as 2D simple rectangular columnar phase of *p2mm* symmetry, Φ_h_ as 2D columnar hexagonal phase of *p6mm* symmetry, Φ_r-c_ as 2D centred rectangular columnar phase with *c2mm* symmetry, k as crystalline phase, and i as isotropic phase.

Dendritic precursors **4b–e** exhibit only a transition between a crystalline structure (k) and an isotropic liquid (i). In contrast, in addition to the aforementioned phase transition, **4a** displayed a 2D periodic array assigned by XRD as a simple rectangular columnar phase (Φ_r-s_) between 34 and 60 °C. These two phase transitions were determined by DSC.

**Fig. 2:**
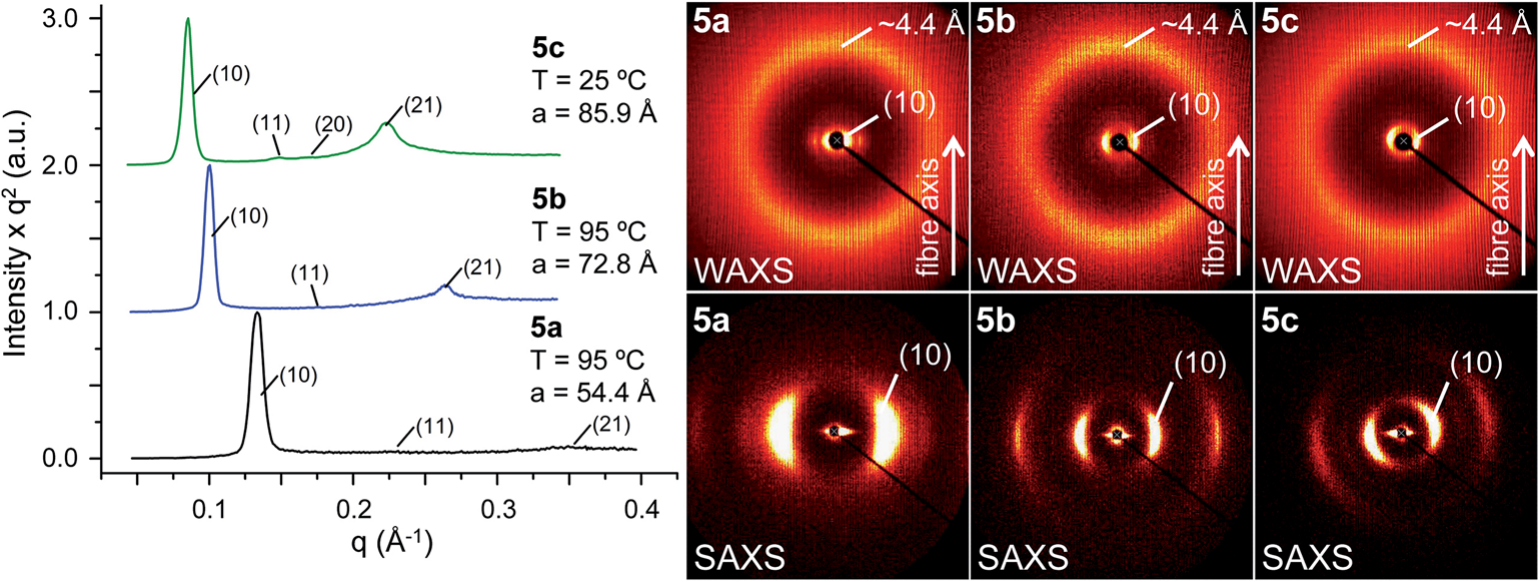
Left: representative small-angle powder diffraction plots of **5a–c** collected in the columnar hexagonal (Φ_h_) phase (collection temperature, diffraction peaks and dimension are indicated). Right: wide- (WAXS) and small- (SAXS) angle X-ray diffraction patterns collected for oriented fibres of **5a–c** in the Φ_h_ phase at 25 °C.

Fullerene derivatives **5a–e** self-organised in 2D periodic arrays. The isotropisation temperatures were determined by DSC. No glass transitions were observed. POM measurements indicated viscous birefringent phases for all compounds. Nevertheless, the optical textures observed on cooling from the isotropic liquids were in general non-characteristic (Fig. S17†). Only compound **5b** displayed a pseudo focal conic fan shaped texture, which is characteristic of a columnar arrangement (Fig. S18†). Unequivocal identification of the mesophases was permitted by XRD. The powder and oriented fibre XRD experiments revealed that all compounds form columnar hexagonal phases (Table 1 and Fig. 2). Analysis of the oriented fibre XRD data shown in Fig. 2 and S20† demonstrated that the columnar hexagonal phases are 2D. Fibre XRD patterns do not exhibit clear features indicative of long range helical features. However, this does not exclude the possibility of helical organization demonstrated by thin film CD data to be discussed later. It is probable that diffuse short range helical features, which are typically observed in dendritic helical columns in the range of 4–5 Å,^18,19^ are smeared out and cannot be separated from the aliphatic chain–chain correlation features observed at 4.4 Å in WAXS (Fig. 2a–c).

**Fig. 3:**
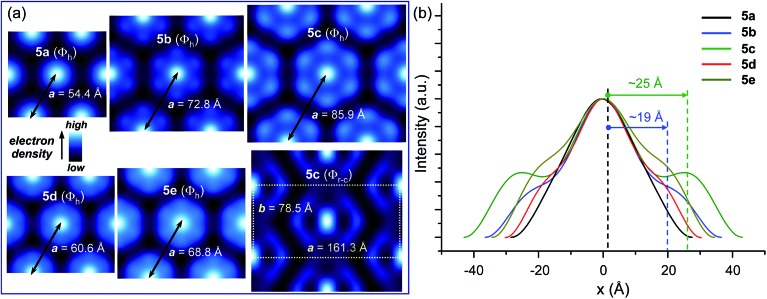
(a) Comparison of the relative electron density distributions calculated from the small-angle powder diffraction data shown in Fig. 2, S19 and S20.† (b) Relative electronic density profiles of the columnar hexagonal (Φ_h_) phases (the estimated distance between the centre of the column and the spacer are indicated).

Interestingly, when the length of the C_60_-dendron spacer increases, the powder XRD profiles exhibit a gradual increase of the relative intensity of the *q*_*hk*_ ≥ *q*_11_ diffraction peaks (Fig. 2). The reconstructed relative electron density maps and their profiles are presented in Fig. 3, S19 and S20.† They indicate that the variation of the powder XRD intensity profile is generated by the increasing length of the C_60_-dendron spacer. This spacer generates a low electron density shell between the aromatic core region and the dendron aromatic groups (Fig. 3). The column diameter follows the trend dictated by the length of the C_60_-dendron spacer (Fig. 3). The dendronized C_60_ with the longest spacer, **5c**, also exhibits an unusual centred rectangular columnar phase (Φ_r-c_) above the low temperature columnar hexagonal phase (Φ_h_) (Fig. 3, S17 and S22† and also Table 2). The reconstructed electron density map of the Φ_r-c_ phase shown in Fig. 3 also displays a shell of low electron density surrounding the C_60_ aromatic core region. In the Φ_r-c_ phase, this variation is reduced in comparison to the Φ_h_ phase, which is most probably due to increased conformational freedom of the structure at higher temperatures that tends to smear out the electron density variations.

**Table 2:** Lattice parameters of the columnar phases of compounds **4a** and **5a–c**

Compound	*T* (°C)	Phase^*a*^	*a*, *b* ^*b*^ (Å)	*d*_10_, *d*_01_, *d*_20_, *d*_31_, *d*_02_ ^*c*^ (Å)
				*d*_20_, *d*_11_, *d*_21_, *d*_31_, *d*_02_, *d*_40_, *d*_22_, *d*_32_, *d*_51_ ^*d*^ (Å)
				*d*_10_, *d*_11_, *d*_20_, *d*_21_ ^*e*^ (Å)
				*d*_20_, *d*_11_, *d*_51_, *d*_42_, *d*_60_ ^*f*^ (Å)
**4a**	20	Φ_r-s_ ^*g*^	63.4, 35.0	63.5, 35.0, 31.8, 18.1, 17.5^*c*^
	50	Φ_r-s_	85.3, 44.2	42.7, 39.3, 30.7, 23.9, 22.1, 21.3, 19.6, 17.5, 15.9^*d*^
**5a**	95	Φ_h_	54.4	47.1, 27.2, —, 17.8^*e*^
**5b**	95	Φ_h_	72.9	63.1, 36.5, —, 23.8^*e*^
**5c**	25	Φ_h_	85.9	74.4, 42.9, 37.2, 28.1^*e*^
	140	Φ_r-c_	161.3, 78.5	80.9, 70.8, 29.9, 28.2, 26.9^*f*^
**5d**	30	Φ_h_	60.6	52.5, 30.3, —, 19.8^*e*^
	75	Φ_h_	60.0	51.9, 30.0, —, 19.6^*e*^
**5e**	30	Φ_h_	68.8	59.7, 34.5, 29.8, 22.5^*e*^

^*a*^Phase notation: Φ_h_ as columnar hexagonal phase, Φ_r-c_ as centered rectangular columnar phase, and Φ_r-s_ as simple rectangular columnar phase.

^*b*^Lattice parameters.

^*c*^ *d*-spacing for the Φ_r-s_ phase.

^*d*^ *d*-spacing for the Φ_r-s_ phase.

^*e*^ *d*-spacing for the Φ_h_ phase.

^*f*^ *d*-spacing for the Φ_r-c_ phase.

^*g*^Phase observed only in the first heating cycle of as-prepared compound.

The reconstructed electron density distributions shown in Fig. 3 were generated based on the (10)+, (11)–, (20)–, and (21)+ diffraction peaks of the 2D Φ_h_ lattice. This phase solution matches the expected electron density variation within the supramolecular columns and was also confirmed by the histograms shown in Fig. S24.† The two peaks of the electron density indicate the expected aliphatic–aromatic microphase segregation. In addition, their relative heights are directly proportional to the number of electrons within their corresponding aromatic region, thereby supporting this phase solution (the histogram of the **5c** exhibits an increased fraction of high electron density region in comparison with **5a**).

**Fig. 4:**
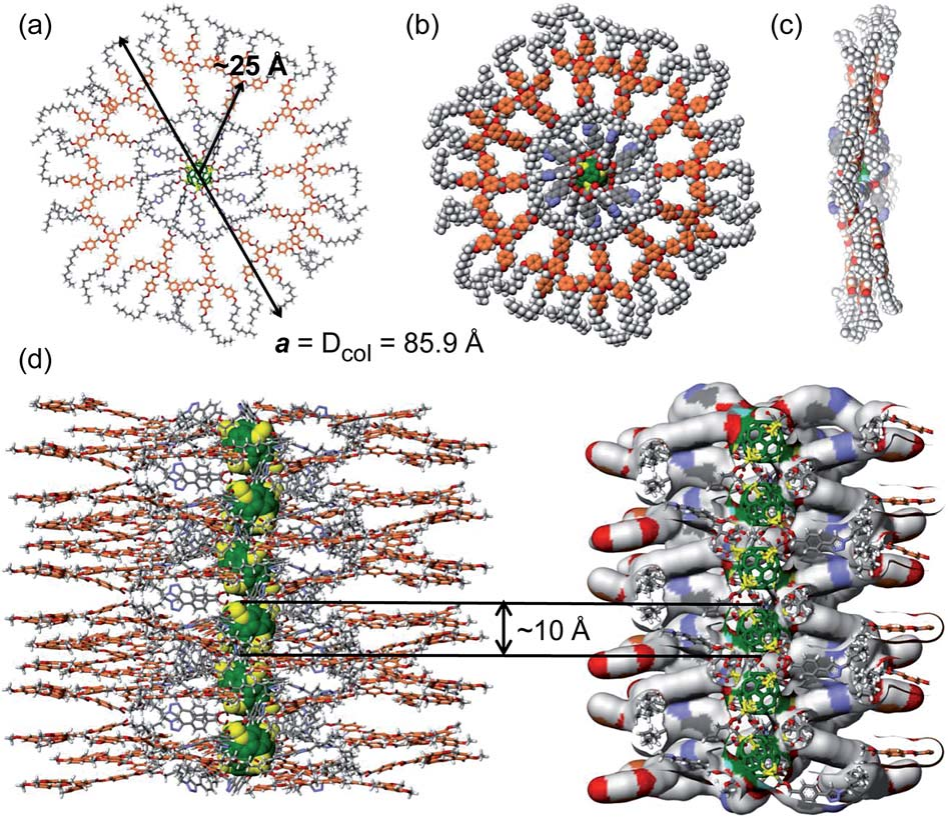
Molecular model of **5c**: (a) top view of the column strata and (b) top and (c) side views of the column strata shown in space filling, and (d) supramolecular organisation model within the columnar phase of dendronized [60]fullerene **5c** showing the 1-dimensional C_60_ nanowire-like structure. Color code: gray as C, white as H, red as O, and blue as N; orange as dendron aromatic rings, green as fullerene core, and yellow as the cyclopropane rings on the fullerene surface.

Based on the electronic density maps reconstructed from XRD, molecular models of the dendronized C_60_ hexakis-adduct were generated. Taking dendronized fullerene **5c** as an example, one molecule forms an entire disc, in which the C_60_ core is confined in the centre and is surrounded by 12 dendrons, forming a ring from which the 36 peripheral alkyl chains radiate outwards (Fig. 4a and b, S23 and S24†). The supramolecular columns are self-organised from these disc-like structures with each disc stratum formed by one single molecule. The calculated disc thickness is about 10 Å, which is approximately equal to the diameter of C_60_. The formation of disc structures constitutes the striking feature for these dodeca-dendronized fullerenes, since one would expect that the quasi-spherical C60 rigid core with an isotropic distribution of dendrons should adopt a globular shape that favours the self-organisation of cubic, tetragonal and quasicrystalline phases.^5^ This result demonstrates that the peripheral dendrons play the predominant role in determining the shape of the supramolecular assembly during structure formation. The reason could be that even if covalently grafted onto the globular core of fullerene, the dendrons still retain their self-assembling properties, forcing their folding towards a disc-shaped structure through the establishment of intramolecular inter-dendron π–π and van der Waals interactions. Other dendronized fullerenes reported here also form Φ_h_ phases, and their lattice parameters are summarized in Table 2.

These structural analysis results support the following self-organisation model. The dendronized fullerene forms supramolecular columns in which each disc contains a single dendronized fullerene molecule. The thickness of the disc is about 10 Å and therefore is equal to the diameter of the fullerene core, which is located at the centre of the disc. The discs are arranged on top of each other at close contact, to form a 1D column of fullerenes in the centre of the supramolecular column (Fig. 4).

### CD experiments

**Fig. 5:**
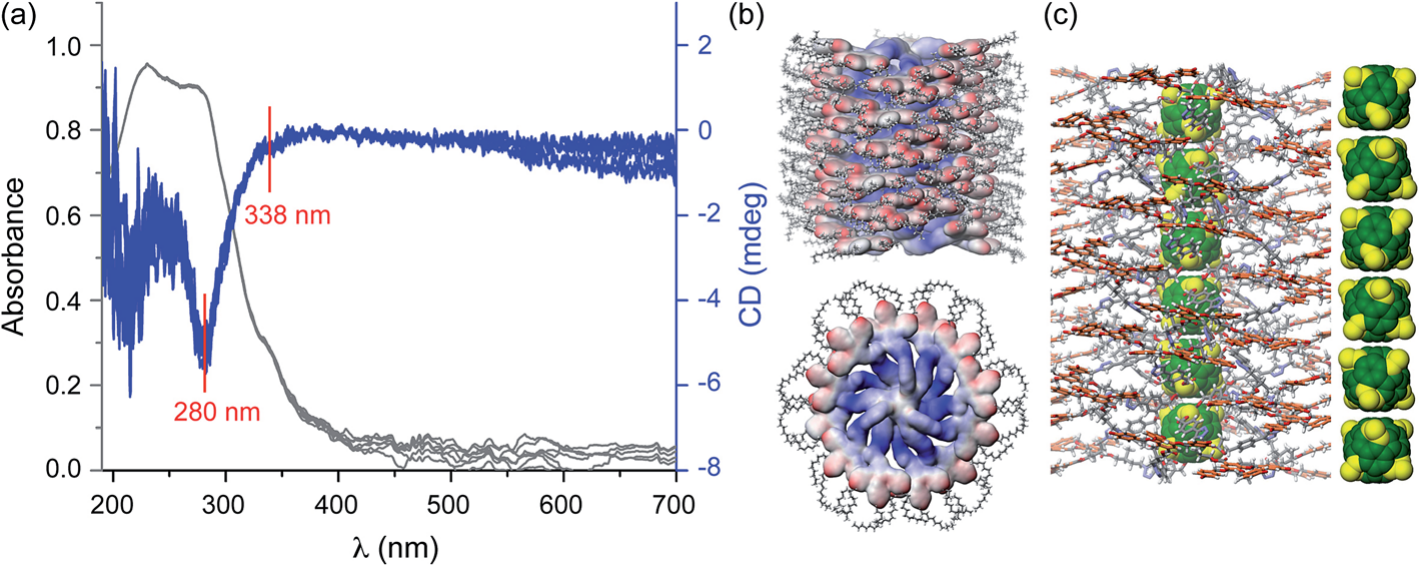
CD/UV spectra measured from the spin coated film of **5d** in the temperature range of 30–70 °C (a), molecular model of the supramolecular column (b) and detail of the core region (c). In (a) the two negative Cotton effects observed at 280 and 338 nm, respectively, are indicated.

The self-assembly of compounds **5d** and **5e**, which both have an enantiopure spacer between the C_60_ core and peripheral dendrons, was investigated by CD spectroscopy, in thin film and in solution. CD spectra of the thin film of **5d** exhibit two weak Cotton effects at 280 nm and 338 nm (Fig. 5a). These two Cotton effects are due to the aromatic part of dendron **5d** and therefore demonstrate that the stereochemical information of the aliphatic stereocentre is transferred to the dendron, which must therefore exhibit a helical conformation in the supramolecular column (Fig. 5b and c). The weakness of the CD signal correlates with the lack of well-defined helical features in wide-angle XRD patterns (Fig. 2a–c), unlike previous systems which exhibit strong CD intensity and clear helical features by XRD.^20^ The CD and XRD data together indicate that the large thickness of the column stratum, dictated by close contact of C_60_ cores, precludes the tightest packing of dendrons into a structure with high intracolumnar helical order.

A mixture of *n*-butanol with methylcyclohexane (7 : 3 v/v) was selected for solution CD studies, since it was able to dissolve **5d** at a concentration of 6.0 × 10^–5^ M. Solution CD studies on this sample (Fig. S25†) showed no emergence of Cotton effects over the temperature range 60 °C to 20 °C, indicating that no self-assembly into helical oligomers occurred under these conditions of solvent and concentration. A very weak positive CD signal was observed at ∼275 nm (Fig. S25b†), but this was invariant with cooling. Cooling below 20 °C resulted in precipitation of the sample.

The CD results in film demonstrate that the supramolecular columns generated from dendronized fullerene are helical and that their helical sense is selected by the stereocentre available in the spacer connecting the dendron to the fullerene.^18^ According to our knowledge this is the first supramolecular helical column containing a 1D fullerene nanowire-like structure in the centre that self-organises into a 2D columnar hexagonal periodic array generated from helical supramolecular columns. Research on the development of similar supramolecular assemblies that self-organise into 3D columnar hexagonal periodic arrays is in progress, as they are of fundamental and technological interest for chiral self-sorting and related applications.^20^

## Conclusions

Dodeca-dendronized fullerene hexa-adducts **5a–e** have been efficiently prepared for the first time from a pre-constructed fullerene hexa-adduct and self-assembling dendritic building blocks *via* click chemistry. These dendronized molecules self-assemble into unprecedented supramolecular discs containing the fullerene at their core. The disc-like conformation adopted by compounds **5a–e** is dictated by the self-assembling capability of the peripheral dendrons. The twelve dendrons drive the conformational equilibrium towards the formation of disc-shaped structures which are perfectly suited for self-organisation into helical 2D columnar hexagonal periodic arrays. XRD investigations of the columnar periodic arrays of **5a–e** together with electron density maps and CD elaborated a structural model for their supramolecular organisation. The fullerene cores are located at the centre of the columns to generate 1D columns of fullerene. The peripheral dendrons of these columns exhibit helicity when the spacer incorporates a stereogenic centre, as demonstrated by CD spectroscopy. However, structures without a stereocentre are also expected to be helical since the stereocentre is only selecting the helical sense of an already helical column.^18,20^ These results provide a synthetically accessible route to a wide range of new fullerene-based helical supramolecular columns that will find utility in organic electronics and other areas of supramolecular science. In addition they provide access to novel supramolecular architectures of great interest for studies of chiral self-sorting in helical complex molecular systems^20^ and demonstrate definitively the self-assembling capabilities of the first generation dendron reported here.
