# Supplementary material for: Self-organisation of dodeca-dendronized fullerene into supramolecular discs and helical columns containing a nanowire-like core
Source: Chem Sci. 2015 Apr 9;6(6):3393–401. doi: 10.1039/c5sc00449g (PMC5657094; doi:10.1039/c5sc00449g)
Supplement: Supplementary file 1 [file SC-006-C5SC00449G-s001.pdf]

Electronic Supplementary Material (ESI) for Chemical Science.
This journal is © The Royal Society of Chemistry 2015

Electronic Supplementary Information (ESI)

Self-organisation of dodeca-dendronized fullerene into supramolecular discs and helical columns containing a nanowire-like core

Sebastiano Guerra,[a] Julien Iehl,[b] Michel Holler,[b] Mihai Peterca,[c] Daniela A. Wilson,[c] Benjamin E. Partridge,[c] Shaodong Zhang,[c] Robert Deschenaux,[*,a] Jean-François Nierengarten[*,b] and Virgil Percec,[*,c]

[a] Institut de Chimie, Université de Neuchâtel, Avenue de Bellevaux 51, 2000 Neuchâtel, Switzerland.

[b] Laboratoire de Chimie des Matériaux Moléculaires, Université de Strasbourg et CNRS, Ecole Européenne de Chimie, Polymères et Matériaux, 25 rue Becquerel, 67087 Strasbourg Cedex 2, France.

[c] Roy & Diana Vagelos Laboratories, Department of Chemistry, University of Pennsylvania, Philadelphia, Pennsylvania 19104-6323, United States.

* Email: robert.deschenaux@unine.ch (R.D.); nierengarten@unistra.fr (J.-F.N.); percec@sas.upenn.edu (V.P.)

Table of Contents

1. Experimental Procedures 2
2. NMR Spectra of Compounds 5a–e 12
Supporting Figure S1 12
Supporting Figure S2 12
Supporting Figure S3 13
Supporting Figure S4 13
Supporting Figure S5 14
Supporting Figure S6 14
Supporting Figure S7 15
Supporting Figure S8 15
Supporting Figure S9 16
Supporting Figure S10 16
3. UV-vis Spectra of Compounds 5a–e 17
Supporting Figure S11 17
Supporting Figure S12 17
Supporting Figure S13 17
Supporting Figure S14 18
Supporting Figure S15 18

4. MALDI-TOF Spectra for Compounds 5b and 5c 19
Supporting Figure S16 19
5. Optical Microscopy on Compounds 5a–c 20
Supporting Figure S17 20
Supporting Figure S18 20
Supporting Figure S19 20
6. XRD Data for Compounds 5a–e 21
Supporting Figure S20 21
Supporting Figure S21 21
Supporting Figure S22 22
7. Molecular Modelling for Compounds 5b–d 23
Supporting Figure S23 23
Supporting Figure S24 23
8. Solution CD Spectra for Compound 5d 24
Supporting Figure S25 24
9. References 25

1. Experimental Procedures

General. Reagents and solvents were purchased as reagent grade and used without further purification. Compounds **5**, **6, 8** and **10** were prepared according to literature procedures.[1,2] THF was distilled over sodium benzophenone ketyl and CH_2Cl_2 over P_2O_5. All reactions were performed in standard glassware under an inert Ar atmosphere. Evaporation and concentration were conducted at water aspirator pressure and drying in vacuo at 10^{-2} Torr. Column chromatography: silica gel 60 (230-400 mesh, 0.040-0.063 mm, E. Merck) or silica gel Brunschwig (63-200, 60 Å). Thin Layer Chromatography (TLC) was performed on glass sheets coated with silica gel 60 F_{254} purchased from E. Merck. IR spectra (cm^{-1}) were recorded on an ATI Mattson Genesis Series FT-IR spectrometer or on a PerkinElmer Spectrum One FT-IR spectrometer. NMR spectra were recorded on a Bruker AC 300 or on a Bruker AV 500 or on a Bruker 400 spectrometer; solvent peaks were used as reference. MALDI-TOF-mass spectra (*m/z*; % relative intensity) were recorded on a Bruker BIFLEX™ matrix-assisted laser desorption time-of-flight mass spectrometer; a saturated solution of 1,8,9-trihydroxyanthracene (dithranol ALDRICH EC: 214-538-0) in CH_2Cl_2 was used as a matrix; ESI-mass spectra were recorded on a Finnigan LCQ electro-spray ionization spectrometer. Elemental analyses were performed by the analytical service at the Laboratoire de Chimie de Coordination, Toulouse (France) or by the Mikroelementar-analytisches Laboratorium ETH-Zürich (Switzerland).

Abbreviations. EDC: *N*-(3-Dimethylaminopropyl)-*N'*-ethylcarbodiimide; DPTS: 4-(dimethylamino)pyridinium *p*-toluenesulfonate; DBU: 1,8-diazabicyclo[5.4.0] undec-7-ene; TBAF: tetrabutylammonium fluoride; ODCB: *o*-dichlorobenzene.

Compound 2. Malonyl chloride (0.95 mL, 9.82 mmol) was added to a solution of **1** (4.02 g, 19.64 mmol), followed by addition of pyridine (1.52 mL, 19.64 mmol) in CH_2Cl_2 (200 mL) at 0 °C. After 1 h the mixture was warmed to room temperature and allowed to stir for 16 h. The reaction mixture was filtered and the solvent of filtrate was evaporated. The crude product was further purified by column chromatography (CH_2Cl_2/hexane 6:4) to yield **2** (2.53 g, 63 %) as a colorless oil. IR (neat): 2175 (C≡C), 1743 (C=O). ^1H NMR (300 MHz, $CDCl_3$): $\delta =$ 7.44 (d, ^3J = 7 Hz, 4 H, Ar-*H*), 7.24 (d, ^3J = 7 Hz, 4 H, Ar-*H*), 5.14 (s, 4 H, OCH_2), 3.47 (s, 2

H, CH_2(CO_2R)$_2$), 0.26 (s, 18 H, $SiMe_3$). ^{13}C NMR (75 MHz, $CDCl_3$): δ = 166.0, 135.4, 132.1, 128.0, 123.3, 104.5, 94.9, 66.7, 41.4.

Compound 3. CBr_4 (3.00 g, 9.02 mmol), **2** (0.43 g, 0.90 mmol), and DBU (0.27 mL, 1.80 mmol) were added successively to a solution of C_{60} (0.065 g, 0.09 mmol) in ODCB (20 mL). The mixture was allowed to stir for 72 h and the solvent was evaporated. The crude product was further purified by column chromatography (CH_2Cl_2/hexane 5:5) to yield **3** (0.18 g, 56 %) as an orange glassy product. IR (neat): 2180 (C≡C), 1748 (C=O). UV/Vis (CH_2Cl_2): 254 (373000), 266 (339700), 278 (sh, 154200), 288 (sh, 111200), 298 (sh, 76500), 319 (sh, 51500), 338 (sh, 37700). ^{1}H NMR (300 MHz, $CDCl_3$): δ = 7.40 (d, ^3J = 8 Hz, 24 H, Ar-*H*), 7.17 (d, ^3J = 8 Hz, 24 H, Ar-*H*), 5.18 (s, 24 H, OCH_2), 0.25 (s, 108 H, $SiMe_3$). ^{13}C NMR (75 MHz, $CDCl_3$): δ = 163.3, 145.9, 140.9, 134.7, 132.1, 128.5, 123.6, 104.6, 95.0, 69.0, 68.1, 45.1. MALDI-TOF-MS: 3569 ([M]$^+$, calcd. for $C_{222}H_{180}O_{24}Si_{12}$: 3568.82).

Compound 4a. A solution of **5**[1] (1.99 g, 1.92 mmol) and NaN_3 (250 mg, 3.84 mmol) in DMF (100 mL) was allowed to stir at 70 °C for 24 h. The mixture was cooled to room temperature and diluted with water (100 mL). The solution was extracted with CH_2Cl_2 (3 × 100 mL). The combined organic phases were washed with water (3 × 100 mL), dried over $MgSO_4$ and concentrated to dryness. The crude product was further purified by precipitation (dissolution in CH_2Cl_2 and precipitation by pouring the solution into MeOH) to yield **4a** as a white solid (1.87 g, 97 %). IR (KBr): 2096 (N_3). ^{1}H NMR (400 MHz, CD_2Cl_2): δ = 7.34 (d, ^3J = 8.5 Hz, 4H, Ar-*H*); 7.23 (d, ^3J = 8.7 Hz, 2H, Ar-*H*); 6.90 (d, ^3J = 8.4 Hz, 4H, Ar-*H*); 6.74 (d, ^3J = 8.9 Hz, 2H, Ar-*H*); 6.62 (s, 2H, Ar-*H*); 5.01 (s, 4H, OCH_2Ar); 4.89 (s, 2H, OCH_2Ar); 4.26 (s, 2H, ArCH_2N_3); 3.97 (t, ^3J = 6.5 Hz, 4H, ArOCH_2); 3.92 (t, ^3J = 6.9 Hz, 2H, ArOCH_2); 1.81-1.71 (m, 6H, ArOCH_2CH_2); 1.48-1.27 (m, 54H, CH_2); 0.88 (t, ^3J = 7.2 Hz, 9H, CH_2CH_3). ^{13}C NMR (100 MHz, CD_2Cl_2): δ = 159.2; 153.2; 130.2; 129.4; 128.9; 114.5; 114.0; 107.5; 71.0; 68.2; 68.1; 55.1; 32.0; 29.77; 29.74; 29.71; 29.70; 29.5; 29.45; 29.4; 26.15; 26.1; 22.8; 14.0. ESI-MS: 1026.73 ([M+Na]$^+$, calcd for $C_{64}H_{97}N_3O_6Na$: 1026.73). Anal. Calcd for $C_{64}H_{97}N_3O_6$: C, 76.53; H, 9.73; N, 4.18; found: C, 76.34; H, 9.52; N, 4.20.

General Procedure for the esterification reactions. EDC (1.2 equiv.) was added to a mixture of **6**[1] (1 equiv.) and the appropriate alcohol or phenol derivative (1 equiv.) in the presence of DPTS (1 equiv.) in dry CH_2Cl_2 at 0 °C. The solution was stirred at room

temperature overnight under Ar, washed with water, dried over $MgSO_4$ and concentrated to dryness.

Compound 4b. Prepared from **6**[1] (690 mg, 0.70 mmol), 1-azidoundecan-11-ol (149 mg, 0.70 mmol), DPTS (206 mg, 0.70 mmol) and EDC (0.11 mL, 0.84 mmol) in CH_2Cl_2 (50 mL). Purification of the residue by column chromatography (CH_2Cl_2) and precipitation (dissolution in CH_2Cl_2 and precipitation by pouring the solution into MeOH) gave pure **4b** as a white solid. IR (KBr): 2096 (N_3), 1709 (CO ester). ^{1}H NMR (400 MHz, CD_2Cl_2): δ = 7.35 (s, 2H, Ar-*H*); 7.34 (d, ^3J = 8.8 Hz, 4H, Ar-H); 7.22 (d, ^3J = 8.8 Hz, 2H, Ar-H); 6.90 (d, ^3J = 8.7 Hz, 4H, Ar-H); 6.75 (d, ^3J = 8.7 Hz, 2H, Ar-H); 5.04 (s, 4H, OCH_2Ar); 4.96 (s, 2H, OCH_2Ar); 4.25 (t, ^3J = 6.5 Hz, 2H, CO_2CH_2); 3.97 (t, ^3J = 6.7 Hz, 4H, OCH_2CH_2); 3.92 (t, ^3J = 6.6 Hz, 2H, OCH_2CH_2); 3.24 (t, ^3J = 7.0 Hz, 2H, CH_2N_3); 1.81-1.71 (m, 8H, OCH_2CH_2 and $CH_2CH_2CO_2$); 1.59-1.55 (m, 2H, $CH_2CH_2N_3$); 1.50-1.27 (m, 68H, CH_2); 0.88 (t, ^3J = 7.0 Hz, 9H, CH_2CH_3). ^{13}C NMR (100 MHz, CD_2Cl_2): δ = 159.3; 152.7; 130.3; 129.5; 128.7; 114.5; 114.1; 108.7; 74.7; 71.0; 68.2; 68.1; 51.6; 32.0; 29.8; 29.73; 29.71; 29.61; 29.59; 29.57; 29.53; 29.52; 29.45; 29.4; 29.2; 28.9; 26.8; 26.1; 22.8; 14.0. ESI-MS: 1210.80 ($[M+Na]^+$, calcd for $C_{75}H_{117}N_3O_8Na$: 1210.87) and 1226.60 ($[M+K]^+$, calcd for $C_{75}H_{117}N_3O_8K$: 1226.85). Anal. Calcd for $C_{75}H_{117}N_3O_8$: C, 75.78; H, 9.92; N, 3.53; found: C, 76.06; H, 9.96; N, 3.33.

Compound 4c. Prepared from **6**[1] (720 mg, 0.73 mmol), **7** (243 mg, 0.73 mmol), DPTS (215 mg, 0.73 mmol) and EDC (0.16 mL, 0.88 mmol) and CH_2Cl_2 (50 mL). The crude product was further purified by column chromatography (CH_2Cl_2) and precipitation (dissolution in CH_2Cl_2 and precipitation by pouring the solution into MeOH) to yield pure **4c** as a white solid (819 mg, 86 %) as a white solid. IR (KBr): 2096 (N_3), 1739 + 1723 (CO ester). ^{1}H NMR (400 MHz, CD_2Cl_2): δ = 8.11 (d, ^3J = 8.7 Hz, 2H, Ar-*H*); 7.52 (s, 2H, Ar-*H*); 7.36 (d, ^3J = 8.5 Hz, 4H, Ar-*H*); 7.30 (d, ^3J = 8.8 Hz, 2H, Ar-*H*); 7.24 (d, ^3J = 8.8 Hz, 2H, Ar-*H*); 6.91 (d, ^3J = 8.7 Hz, 4H, Ar-*H*); 6.76 (d, ^3J = 8.8 Hz, 2H, Ar-*H*); 5.08 (s, 4H, OCH_2Ar); 5.02 (s, 2H, OCH_2Ar); 4.31 (t, ^3J = 6.5 Hz, 2H, CO_2CH_2); 3.97 (t, ^3J = 6.8 Hz, 4H, OCH_2CH_2); 3.93 (t, ^3J = 6.6 Hz, 2H, OCH_2CH_2); 3.25 (t, ^3J = 7.0 Hz, 2H, CH_2N_3); 1.81-1.73 (m, 8H, OCH_2CH_2 and $CH_2CH_2CO_2$); 1.60-1.55 (m, 2H, $CH_2CH_2N_3$); 1.50-1.28 (m, 68H, CH_2); 0.88 (t, ^3J = 7.0 Hz, 9H, CH_2CH_3). ^{13}C NMR (100 MHz, CD_2Cl_2): δ = 165.8; 164.3; 159.4; 159.2; 154.7; 154.4; 152.9; 143.1; 131.1; 130.3; 129.5; 129.45; 128.5; 128.4; 124.0; 121.9; 114.5; 114.1; 109.5; 74.8; 71.2; 68.2; 68.1; 65.3; 51.6; 32.0; 29.77; 29.74; 29.72; 29.7; 29.69; 29.58; 29.57; 29.55;

29.52; 29.45; 29.43; 29.42; 29.4; 29.35; 29.2; 28.9; 28.8; 26.8; 26.15; 26.14; 26.13; 22.8; 14.0. ESI-MS: 1330.67 ($[M+Na]^+$, calcd for $C_{82}H_{121}N_3O_{10}Na$: 1330.90). Anal. Calcd for $C_{82}H_{121}N_3O_{10}$: C, 75.25; H, 9.32; N, 3.21; found: C, 75.27; H, 9.46; N, 3.08.

Compound 4d. Prepared from **6**[1] (1.50g, 1.51 mmol), **9** (170 mg, 1.51 mmol), DPTS (440 mg, 1.51 mmol) and EDC (0.32 mL, 1.81 mmol) and CH_2Cl_2 (50 mL). The crude product was further purified by column chromatography (CH_2Cl_2) and precipitation (dissolution in CH_2Cl_2 and precipitation by pouring the solution into EtOH) to yield pure **4d** as a white solid. IR (KBr): 2097 (N_3), 1702 + 1614 (CO ester). ^{1}H NMR (400 MHz, CD_2Cl_2): δ = 7.34 (d, ^3J = 8.8 Hz, 4H, Ar-*H*); 7.33 (s, 2H, Ar-*H*); 7.23 (d, ^3J = 8.6 Hz, 2H, Ar-*H*); 6.90 (d, ^3J = 8.6 Hz, 4H, Ar-*H*); 6.75 (d, ^3J = 8.8 Hz, 4H, Ar-*H*); 5.05 (s, 4H, ArOCH_2); 4.98 (s, 2H, ArOCH_2); 4.19 (m, 2H, C$H_2$$O_2$C); 3.96 (t, ^3J = 6.7 Hz, 4H, OCH_2); 3.92 (t, ^3J = 6.6 Hz, 2H, OCH_2); 3.36 (m, 2H, CH_2N_3); 2.20 (m, 1H, C*H*); 1.81-1.72 (m, 6H, OCH_2CH_2); 1.49-1.42 (m, 6H, O$CH_2$$CH_2CH_2$); 1.35-1.27 (m, 48H, C$H_2$); 1.06 (d, ^3J = 6.9 Hz, 3H, CHCH_3); 0.88 (t, ^3J = 7.2 Hz, 9H, CH_3). ^{13}C NMR (100 MHz, CD_2Cl_2): δ = 166.3; 159.8; 159.7; 153.2; 142.9; 130.8; 130.1; 129.9; 129.1; 125.6; 115.0; 114.6; 109.3; 75.2; 71.5; 68.69; 68.6; 67.2; 55.1; 33.9; 32.5; 30.26; 30.23; 30.2; 30.19; 30.03; 30.01; 29.94; 29.9; 29.88; 26.6; 23.3; 15.1; 14.4. ESI-MS: 1112.80 ($[M+Na]^+$, calcd for $C_{68}H_{103}N_3O_8Na$: 1112.76). Anal. Calcd for $C_{68}H_{103}N_3O_8$: C, 74.89; H, 9.52; N, 3.85; found: C, 75.09; H, 9.48; N, 3.72.

Compound 4e. Prepared from **6**[1] (1.80 g, 1.81 mmol), **12** (430 mg, 1.81 mmol), DPTS (530 mg, 1.81 mmol) and EDC (0.39 mL, 2.17 mmol) and CH_2Cl_2 (50 mL). The crude product was further purified by column chromatography (CH_2Cl_2) and precipitation (dissolution in CH_2Cl_2 and precipitation by pouring the solution into MeOH) to yield pure **4e** as a white solid. IR (KBr): 2100 (N_3). ^{1}H NMR (400 MHz, CD_2Cl_2): δ = 8.12 (d, ^3J = 8.8 Hz, 2H, Ar-*H*); 7.52 (s, 2H, Ar-*H*); 7.36 (d, ^3J = 8.6 Hz, 4H, Ar-*H*); 7.31 (d, ^3J = 8.7 Hz, 2H, Ar-*H*); 7.24 (d, ^3J = 8.5 Hz, 2H, Ar-*H*); 6.91 (d, ^3J = 8.7 Hz, 4H, Ar-*H*); 6.76 (d, ^3J = 8.7 Hz, 2H, Ar-*H*); 5.08 (s, 4H, ArOCH_2); 5.02 (s, 2H, ArOCH_2); 4.28 (m, 2H, C$H_2$$O_2$C); 3.97 (t, ^3J = 6.6 Hz, 4H, OCH_2); 3.92 (t, ^3J = 6.7 Hz, 2H, OCH_2); 3.42 (t, ^3J = 6.1 Hz, C$H_2$$N_3$); 2.24 (m, 1H, C*H*); 1.81-1.73 (m, 6H, OCH_2CH_2); 1.49-1.41 (m, 6H, O$CH_2$$CH_2CH_2$); 1.35-1.27 (m, 48H, C$H_2$); 1.10 (d, ^3J = 7.0 Hz, 3H, CHCH_3); 0.88 (t, ^3J = 7.0 Hz, 9H, CH_3). ^{13}C NMR (100 MHz, CD_2Cl_2): δ = 166.0; 164.8; 159.8; 159.7; 155.4; 153.4; 143.6; 130.8; 130.1; 129.9; 129.1; 125.6; 115.0; 114.6; 109.3; 75.2; 71.5; 68.7; 68.6; 67.2; 55.1; 33.9; 32.5; 30.26; 30.23; 30.2; 30.19; 30.03;

30.01; 29.94; 29.9; 29.88; 26.6; 23.3; 15.1; 14.4. ESI-MS: 1232.90 ($[M+Na]^+$, calcd for $C_{75}H_{107}N_3O_{10}Na$: 1232.79). Anal. Calcd for $C_{75}H_{107}N_3O_{10}$: C, 74.41; H, 8.91; N, 3.47; found: C, 74.47; H, 8.76; N, 3.42.

General Procedure for the click reactions. $CuSO_4\cdot 5H_2O$ (0.1 equiv.) and sodium ascorbate (0.3 equiv.) were added to a mixture of **3** (1 equiv.) and the corresponding azide (13 equiv.) in CH_2Cl_2/H_2O (1:1). TBAF (14 equiv. in THF) was added and the resulting mixture was vigorously stirred at room temperature for 12 h under N_2. The organic layer was diluted with CH_2Cl_2, washed with water, dried over $MgSO_4$ and concentrated. The product was then purified as outlined in the following text.

Compound 5a. Compounds **3** (76 mg, 0.021 mmol), **4a** (280 mg, 0.28 mmol), $CuSO_4\cdot 5H_2O$ (0.34 mg, 0.0021 mmol), sodium ascorbate (1.2 mg, 0.006 mmol), and TBAF (0.27 mL, 0.27 mmol) were dissolved in CH_2Cl_2/H_2O (1:1, 1 mL). The reaction mixture was purified by column chromatography (CH_2Cl_2 containing 2% of methanol) followed by gel permeation chromatography (Biobeads SX-1, CH_2Cl_2) to yield **5a** (64 mg, 64 %) as an orange glassy product. IR (neat): 1744 (C=O). UV-vis (CH_2Cl_2): 255 (471200), 275 (sh, 327300), 282 (sh, 245700), 321 (sh, 63200), 337 (sh, 48900). ^{1}H NMR ($CDCl_3$, 300 MHz): δ = 7.77 (s, 12H, *br*, Triazole-*H*), 7.68 (d, ^3J = 8.0 Hz, 24H, Ar-*H*), 7.25-7.17 (m, 96H, Ar-*H*), 6.81 (d, ^3J = 8.0 Hz, 48H, Ar-*H*), 6.72 (d, ^3J = 8.0 Hz, 24H, Ar-*H*), 6.60 (s, 24H, Ar-*H*), 5.33 (s, 24H, *br*, OCH_2Ar), 5.20 (s, 24H, OCH_2Ar), 4.96-4.89 (m, 48H, OCH_2Ar), 4.88-4.83 (m, 24H, OCH_2Ar), 3.94-3.84 (m, 72H, $ArOCH_2$), 1.80-1.70 (m, 72H, $ArOCH_2CH_2$), 1.50-1.20 (m, 648H, CH_2), 0.88 (m, 108H, CH_2CH_3). ^{13}C NMR ($CDCl_3$, 100 MHz): δ = 163.4, 158.95, 158.9, 153.3, 147.4, 146.0, 142.9, 141.0, 138.7, 134.2, 130.8, 130.1, 129.7, 129.4, 129.2, 129.1, 128.5, 126.5, 126.3, 125.7, 120.4, 114.3, 114.0, 107.7, 74.8, 71.0, 69.1, 68.3, 68.0, 54.1, 45.2, 31.9, 29.7, 29.6, 29.4, 29.3, 26.0, 22.7, 14.1. Anal. Calcd for $C_{972}H_{1248}N_{36}O_{96}\cdot 2CH_2Cl_2$: C 77.26, H 8.33, N 3.33; found: C 77.14, H 8.03, N 3.15.

Compound 5b. Compounds **3** (45 mg, 0.012 mmol), **4b** (195 mg, 0.16 mmol), $CuSO_4\cdot 5H_2O$ (0.2 mg, 0.0014 mmol), sodium ascorbate (0.75 mg, 0.004 mmol), and TBAF (0.18 mL, 0.18 mmol) were dissolved in CH_2Cl_2/H_2O (1:1, 1 mL). The reaction mixture was purified by column chromatography (CH_2Cl_2 containing 2% of methanol) followed by gel permeation chromatography (Biobeads SX-1, CH_2Cl_2) to yield **5b** (181 mg, 85 %) as an orange glassy product. IR (neat): 1744 (C=O), 1713 (C=O). UV-vis (CH_2Cl_2): 262 (449700), 275 (sh,

368400), 283 (sh, 269600), 307 (sh, 88700), 337 (sh, 35600); ^{1}H NMR ($CDCl_3$, 300 MHz): δ = 7.84 (s, 12H, *br*, Triazole-*H*), 7.70 (d, ^3J = 8.0 Hz, 24H, Ar-*H*), 7.35 (s, 24H, Ar-*H*), 7.32 (m, 72H, Ar-*H*), 7.23 (d, ^3J = 8.0 Hz, 24H, Ar-*H*), 6.87 (d, ^3J = 8.0 Hz, 48H, Ar-*H*), 6.74 (d, ^3J = 8.0 Hz, 24H, Ar-*H*), 5.24 (s, 24H, *br*, OCH_2Ar), 5.02 (s, 48H, OCH_2Ar), 4.98 (s, 24H, OCH_2Ar), 4.40-4.30 (m, 24H, CH_2O_2CAr), 4.25 (t, ^3J = 6.0 Hz, 24H, Triazole-CH_2), 3.94 (t, ^3J = 6.0 Hz, 48H, $ArOCH_2$), 3.90 (t, ^3J = 6.0 Hz, 24H, $ArOCH_2$), 2.00-1.86 (m, 24H, $CH_2CH_2O_2CAr$), 1.82-1.68 (m, 96H, Triazole-CH_2CH_2 and $ArOCH_2CH_2$), 1.50-1.20 (m, 816H, CH_2), 0.87 (t, ^3J = 6.0 Hz, 108H, CH_2CH_3). ^{13}C NMR ($CDCl_3$, 100 MHz): δ = 166.2, 163.5, 159.0, 158.9, 152.6, 147.0, 146.0, 142.5, 141.1, 134.1, 131.0, 130.2, 129.5, 129.3, 129.1, 128.6, 125.8, 125.3, 120.1, 114.4, 114.1, 109.2, 74.7, 71.1, 69.1, 68.0, 67.9, 65.1, 54.1, 50.4, 45.2, 43.4, 31.9, 30.4, 30.1, 29.7, 29.65, 29.6, 29.55, 29.5, 29.45, 29.4, 29.35, 29.3, 29.2, 29.1, 28.7, 26.9, 26.6, 26.1, 26.0, 22.7, 14.1. MALDI-TOF-MS: 16970 ($[MH]^+$, calcd. for $C_{1086}H_{1489}N_{36}O_{120}$: 16968.65). Anal. Calcd for $C_{1086}H_{1488}N_{36}O_{120}$: C 76.87, H 8.84, N 2.97; found: C 77.34, H 8.62, N 2.81.

Compound 5c. Compounds **3** (42 mg, 0.012 mmol), **4c** (200 mg, 0.15 mmol), $CuSO_4 \cdot 5H_2O$ (0.2 mg, 0.0014 mmol), sodium ascorbate (0.75 mg, 0.004 mmol), and TBAF (0.16 mL, 0.16 mmol) were dissolved in CH_2Cl_2/H_2O (1:1, 1 mL). The reaction mixture was purified by column chromatography (CH_2Cl_2 containing 1% of methanol) followed by gel permeation chromatography (Biobeads SX-1, CH_2Cl_2) to yield **4c** (192 mg, 89%) as an orange glassy product. IR (neat): 1737 (C=O), 1718 (C=O). UV-vis (CH_2Cl_2): 275 (sh, 489600), 282 (sh, 413800), 308 (sh, 141900), 336 (sh, 44500). ^{1}H NMR ($CDCl_3$, 300 MHz): δ = 8.12 (d, ^3J = 8.0 Hz, 24H, Ar-*H*), 7.87 (s, 12H, *br*, Triazole-*H*), 7.72 (d, ^3J = 8.0 Hz, 24H, Ar-*H*), 7.51 (s, 24H. Ar-*H*), 7.34 (d, ^3J = 8.0 Hz, 48H, Ar-*H*), 7.30-7.24 (m, 72H, Ar-*H*), 6.89 (d, ^3J = 8.0 Hz, 48H, Ar-*H*), 6.77 (d, ^3J = 8.0 Hz, 24H, Ar-*H*), 5.25 (s, 24H, *br*, OCH_2Ar), 5.07 (s, 48H, OCH_2Ar), 5.05 (s, 24H, OCH_2Ar), 4.42-4.35 (m, 24H, CH_2O_2CAr), 4.32 (t, ^3J = 6.0 Hz, 24H, Triazole-CH_2), 3.96 (t, ^3J = 6.0 Hz, 48H, $ArOCH_2$), 3.91 (t, ^3J = 6.0 Hz, 24H, $ArOCH_2$), 2.00-1.90 (m, 24H, $CH_2CH_2O_2CAr$), 1.84-1.70 (m, 96H, Triazole-CH_2CH_2 and $ArOCH_2CH_2$), 1.50-1.20 (m, 816H, CH_2), 0.89 (t, ^3J = 6.0 Hz, 108H, CH_2CH_3). ^{13}C NMR ($CDCl_3$, 75 MHz): δ = 165.8, 164.2, 163.5, 159.1, 159.0, 154.6, 152.8, 147.0, 146.0, 143.4, 141.0, 138.1, 134.2, 131.1, 130.2, 129.35, 129.3, 129.1, 128.4, 128.1, 125.8, 123.7, 121.7, 120.1, 114.5, 114.1, 109.9, 74.7, 71.2, 69.1, 68.3, 68.05, 68.0, 65.2, 50.4, 45.3, 31.9, 30.3, 29.65, 29.6, 29.55, 29.5, 29.45, 29.4, 29.35, 29.3, 29.0, 28.7, 26.5, 26.0, 25.9, 22.6, 14.0. MALDI-TOF-MS: 18408

([M]$^+$, calcd. for $C_{1170}H_{1536}N_{36}O_{144}$: 18408.91). Anal. Calcd for $C_{1170}H_{1536}N_{36}O_{144}$: C, 76.34; H, 8.41; N, 2.74; found: C, 76.34; H, 8.80; N, 2.65.

Compound 5d. Compounds **3** (35 mg, 0.0098 mmol), **4d** (140 mg, 0.13 mmol), $CuSO_4{\cdot}5H_2O$ (0.16 mg, 0.001 mmol), sodium ascorbate (0.6 mg, 0.003 mmol) and TBAF (0.14 mL, 0.14 mmol) in CH_2Cl_2/H_2O (1:1, 1 mL). The reaction mixture was purified by column chromatography (CH_2Cl_2 containing 1% of methanol) followed by gel permeation chromatography (Biobeads SX-1, CH_2Cl_2) to yield **5d** (98 mg, 63%) as an orange glassy product. IR (neat): 1744 (C=O), 1716 (C=O). UV-vis (CH_2Cl_2): 263 (550300), 275 (sh, 475300), 282 (sh, 370900), 297 (sh, 169300), 338 (sh, 49900). ^{1}H NMR ($CDCl_3$, 300 MHz): δ = 7.98 (s, 12H, *br*, Triazole-*H*), 7.78-7.65 (m, 24H, Ar-*H*), 7.34 (s, 24H, Ar-*H*), 7.32-7.18 (m, 96H, Ar-*H*), 6.90-6.80 (m, 48H, Ar-*H*), 6.78-6.70 (m, 24H, Ar-*H*), 5.30-5.15 (m, 24H, OC*H*$_2$Ar), 5.05-4.90 (m, 72H, OC*H*$_2$Ar), 4.52-4.40 (m, 12H, Triazole-C*H*$_2$), 4.38-4.15 (m, 36H, Triazole-C*H*$_2$ and C*H*$_2$O$_2$CAr), 3.95-3.85 (m, 72H, ArOC*H*$_2$), 2.70-2.54 (m, 12H, C*H*), 1.80-1.68 (m, 72H, ArOCH$_2$C*H*$_2$), 1.50-1.18 (m, 648H, C*H*$_2$), 1.01 (m, 36H, CHC*H*$_3$), 0.87 (t, ^3J = 6.0 Hz, 108H, CH$_2$C*H*$_3$). ^{13}C NMR ($CDCl_3$, 100 MHz): δ = 165.9, 163.4, 159.0, 152.6, 147.2, 146.0, 142.9, 141.0, 138.7, 134.3, 134.1, 130.8, 130.4, 130.2, 129.4, 129.2, 128.9, 128.5, 126.5, 125.9, 124.6, 121.0, 120.4, 114.4, 114.1, 109.3, 74.7, 71.1, 69.1, 68.3, 68.0, 67.9, 66.5, 66.3, 53.4, 53.1, 45.2, 34.3, 31.9, 29.65, 29.6, 29.4, 29.3, 26.0, 14.6, 14.1. Anal. Calcd for $C_{1002}H_{1320}N_{36}O_{120}$: C, 76.22; H, 8.43; N, 3.19; found: C, 76.08; H, 8.25; N, 2.82.

Compound 5e. Compounds **3** (30 mg, 0.0084 mmol), **4e** (133 mg, 0.11 mmol), $CuSO_4{\cdot}5H_2O$ (0.13 mg, 0.0008 mmol), sodium ascorbate (0.5 mg, 0.0025 mmol), and TBAF (0.12 mL, 0.12 mmol) were dissolved in CH_2Cl_2/H_2O (1:1, 0.5 mL). The reaction mixture was purified by column chromatography (CH_2Cl_2 containing 2% of methanol) followed by gel permeation chromatography (Biobeads SX-1, CH_2Cl_2) to yield **5e** (90 mg, 62%) as an orange glassy product. IR (neat): 1733 (C=O); UV-vis (CH_2Cl_2): 275 (sh, 516800), 282 (sh, 435900), 297 (sh, 225000), 336 (sh, 49600). ^{1}H NMR ($CDCl_3$, 300 MHz): δ = 8.11 (d, ^3J = 8.0 Hz, 24H, Ar-*H*), 8.01 (s, 12H, Triazole-*H*), 7.80-7.68 (m, 24H, Ar-*H*), 7.49 (s, 24H, Ar-*H*), 7.36-7.20 (m, 120H, Ar-*H*), 6.88 (d, ^3J = 8.0 Hz, 48H, Ar-*H*), 6.74 (d, ^3J = 8.0 Hz, 24H, Ar-*H*), 5.32-5.15 (m, 24H, OC*H*$_2$Ar), 5.08-4.98 (m, 72H, OC*H*$_2$Ar), 4.60-4.45 (m, 12H, Triazole-C*H*$_2$), 4.40-4.20 (m, 36H, Triazole-C*H*$_2$ and C*H*$_2$O$_2$CAr), 4.00-3.85 (m, 72H, ArOC*H*$_2$), 2.72-2.55 (m, 12H, C*H*), 1.82-1.70 (m, 72H, ArOCH$_2$C*H*$_2$), 1.50-1.20 (m, 648H, C*H*$_2$), 1.07 (m, 36H,

CHCH_3), 0.87 (t, ^3J = 6.0 Hz, 108H, CH_2CH_3). ^{13}C NMR ($CDCl_3$, 100 MHz): δ = 165.5, 164.2, 163.4, 159.1, 159.0, 154.9, 152.8, 147.2, 145.9, 143.4, 142.9, 141.0, 138.7, 134.3, 134.1, 131.2, 130.8, 130.2, 129.3, 129.1, 128.9, 128.3, 127.4, 126.5, 125.9, 123.6, 121.9, 121.1, 120.5, 114.4, 114.1, 109.8, 74.8, 71.2, 69.0, 68.3, 68.0, 67.9, 66.5, 66.4, 66.3, 53.0, 45.2, 34.3, 31.9, 29.7, 29.65, 29.6, 29.4, 29.35, 29.3, 26.0, 22.6, 14.6, 14.1. Anal. Calcd for $C_{1086}H_{1368}N_{36}O_{144}$: C, 75.70; H, 8.00; N, 2.93; found: C, 75.47; H, 7.84; N, 2.93.

Compound 9. A solution of *R*-(-)-3-bromo-2-methyl-1-propanol (2.00 g, 13.07 mmol) and NaN_3 (1.70 g, 26.14 mmol) in DMF (50 mL) was allowed to stir at 70 °C for 24 h. The mixture was cooled to room temperature and diluted with water (100 mL). The solution was extracted with CH_2Cl_2 (3 × 100 mL). The combined organic phases were washed with water (3 × 100 mL), dried over $MgSO_4$ and concentrated to afford compound **9** (0.64 g, 43 %) as a colorless oil. ^{1}H NMR (400 MHz, $CDCl_3$): δ = 3.57 (m, 2H, CH_2OH); 3.34 (m, 2H, CH_2N_3); 1.94 (m, 1H, C*H*); 0.97 (d, ^3J = 6.9 Hz, 3H, CHCH_3). ^{13}C NMR (100 MHz, $CDCl_3$): δ = 65.5; 54.9; 36.1; 14.7.

Compound 11. A solution of **10**[2] (1.41 g, 5.57 mmol), **9** (640 mg, 5.57 mmol) and DPTS (1.64 g, 5.57 mmol) in dry CH_2Cl_2 (50 mL) was cooled at 0 °C, and EDC (1.18 ml, 6.68 mmol) was added. The solution was then allowed to stir overnight at room temperature. The reaction mixture was washed with water, dried over $MgSO_4$ and concentrated to dryness. The crude product was further purified by column chromatography (CH_2Cl_2) to yield **11** (1.11 g, 57 %) as a colorless oil. ^{1}H NMR (400 MHz, $CDCl_3$): δ = 7.94 (d, ^3J = 8.7 Hz, 2H, Ar-*H*); 6.87 (d, ^3J = 8.8 Hz, 2H, Ar-*H*); 4.22 (m, 2H, CH_2O_2C); 3.38 (m, 2H, CH_2N_3,); 2.21 (m, 1H, C*H*); 1.09 (d, ^3J = 6.9 Hz, 3H, CHCH_3); 0.99 (s, 9H, ArOSiC(CH_3)$_3$); 0.23 (s, 6H, ArOSi(CH_3)$_2$). ^{13}C NMR (400 MHz, $CDCl_3$): δ = 166.4; 160.4; 131.8; 123.2; 120.1; 66.5; 54.7; 33.7; 25.8; 18.5; 15.0, -4.2. ESI-MS: 372.30 ($[M+Na]^+$, cacld for $C_{17}H_{27}N_3O_3SiNa$: 372.17).

Compound 12. A solution of $Zn(BF_4)_2$·6-7 H_2O (4.02 g, 16.82 mmol) in water (10 mL) was added to a solution of **11** (840 mg, 2.40 mmol) in THF (90 mL). The resulting mixture was stirred at 50 °C for 24 h. The mixture was concentrated and the precipitate filtered and washed with water to give **12** (260 mg, quantitative) as a colorless oil. ^{1}H NMR (400 MHz, $CDCl_3$): δ = 7.95 (d, ^3J = 8.9 Hz, 2H, Ar-*H*); 6.88 (d, ^3J = 8.9 Hz, 2H, Ar-*H*); 6.31 (bs, 1H,

O*H*); 4.23 (m, 2H, CH_2O_2C); 3.38 (m, 2H, CH_2N_3); 2.22 (m, 1H, C*H*); 1.09 (d, ^3J = 6.8 Hz, 3H, CHCH_3). ^{13}C NMR (100 MHz, $CDCl_3$): δ = 165.5; 159.4; 131.1; 121.4; 114.4; 65.6; 53.6; 32.6; 13.9. ESI-MS: 234.10 ($[M-H]^-$, calcd for $C_{11}H_{12}N_3O_3$: 234.09).

2. NMR Spectra of Compounds 5a–e

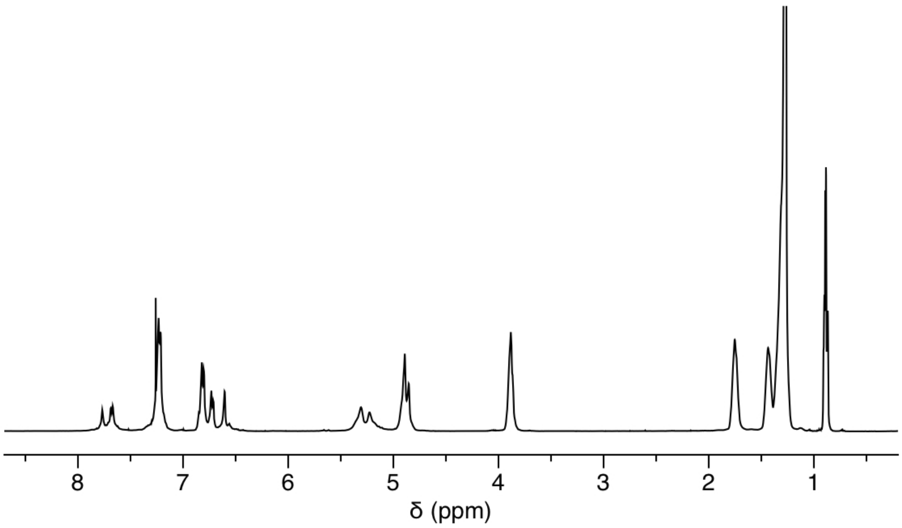

Fig. S1 ^{1}H NMR spectrum of compound **5a** ($CDCl_3$, 400 MHz).

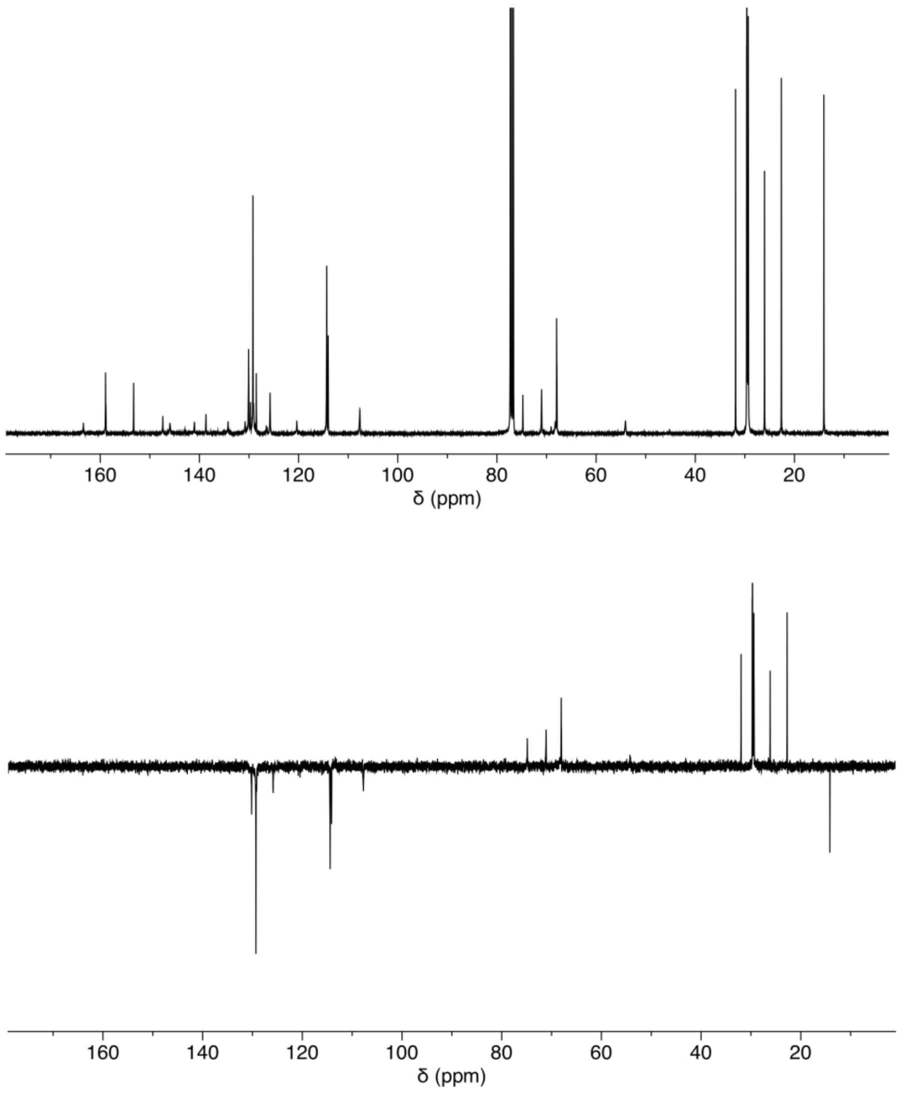

Fig. S2 ^{13}C NMR (top) and DEPT (bottom) spectra of compound **5a** ($CDCl_3$, 100 MHz).

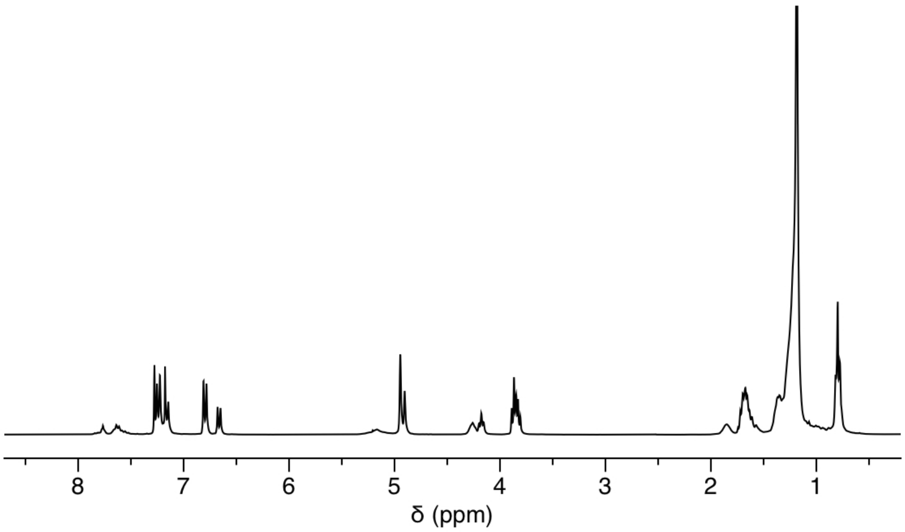

Fig. S3 ^{1}H NMR spectrum of compound **5b** ($CDCl_3$, 400 MHz).

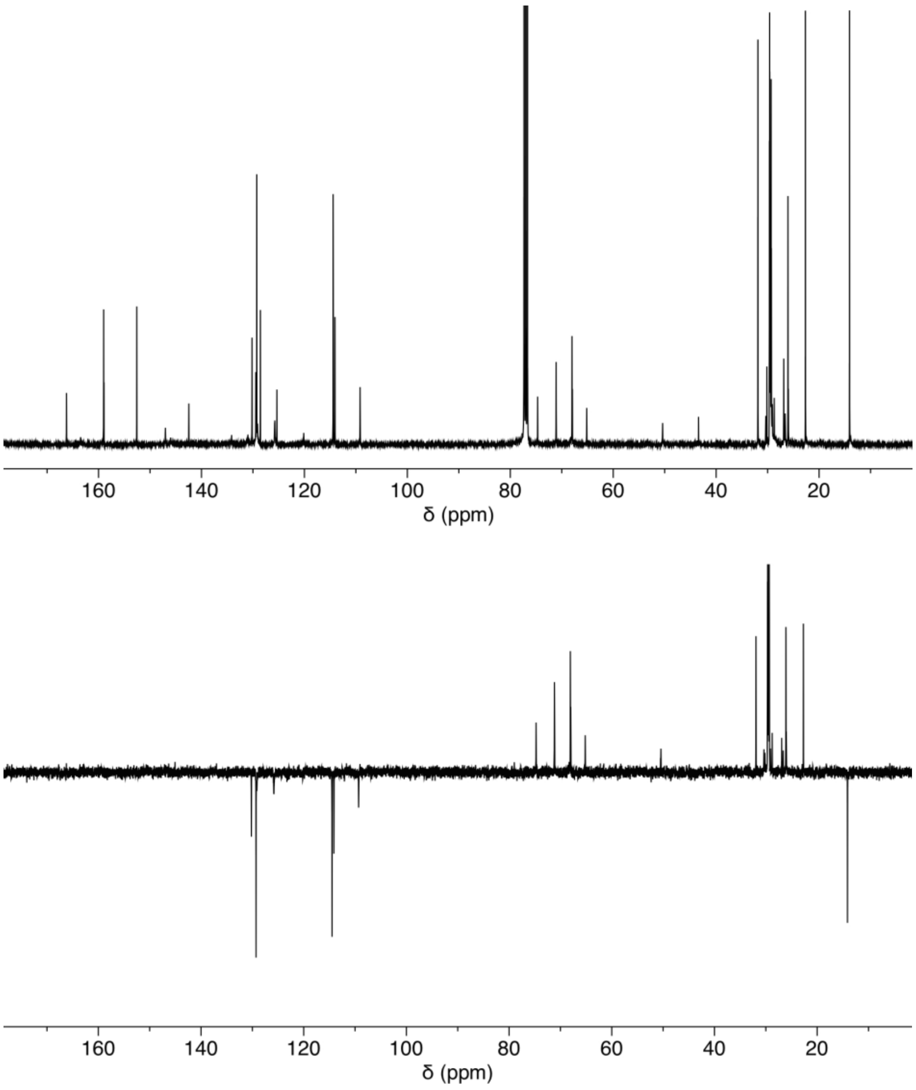

Fig. S4 ^{13}C NMR (top) and DEPT (bottom) spectra of compound **5b** ($CDCl_3$, 100 MHz).

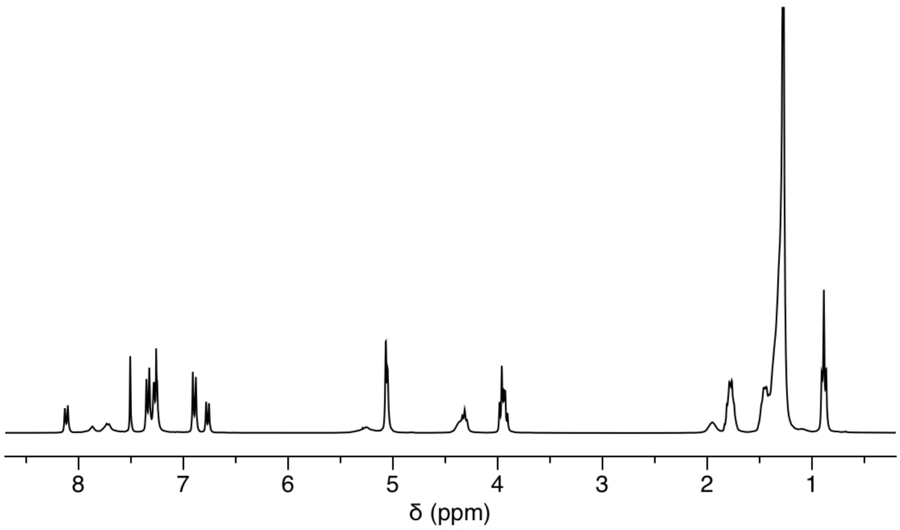

Fig. S5 ^{1}H NMR spectrum of compound **5c** ($CDCl_3$, 400 MHz).

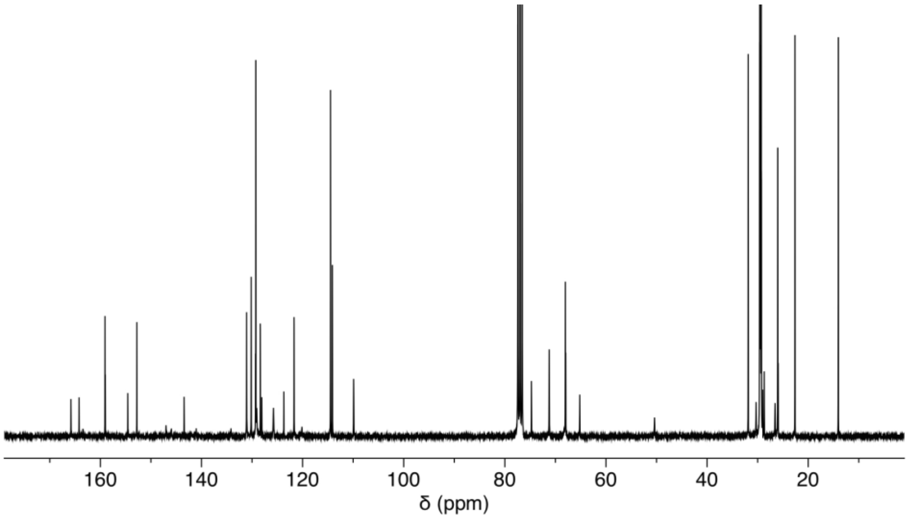

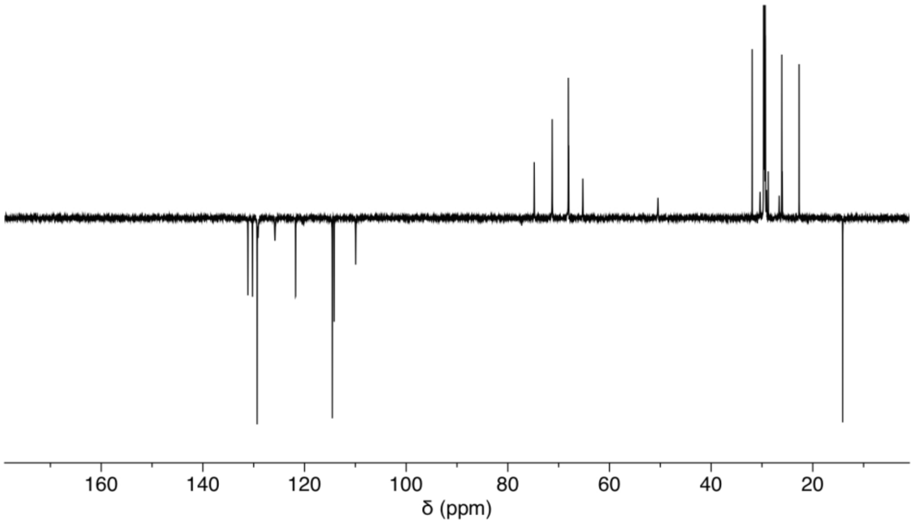

Fig. S6 ^{13}C NMR (top) and DEPT (bottom) spectra of compound **5c** ($CDCl_3$, 100 MHz).

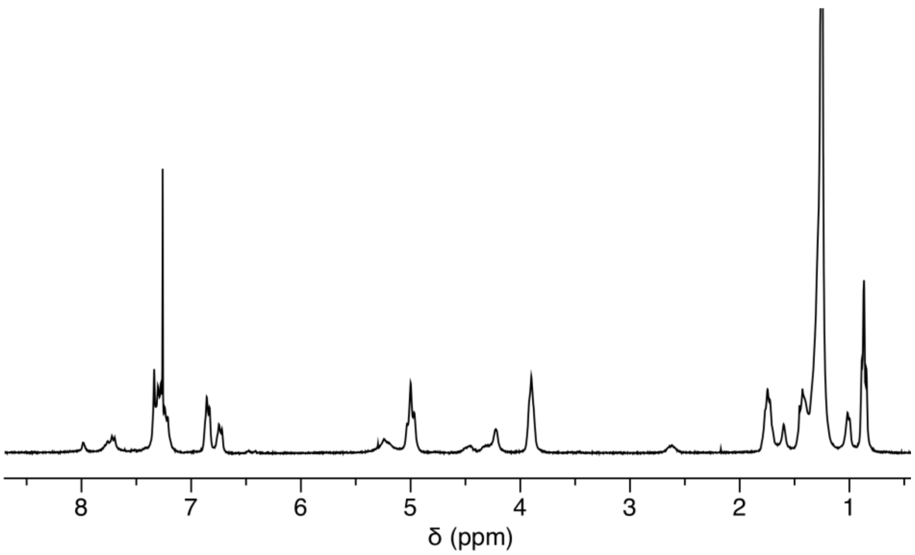

Fig. S7 ^{1}H NMR spectrum of compound **5d** ($CDCl_3$, 400 MHz).

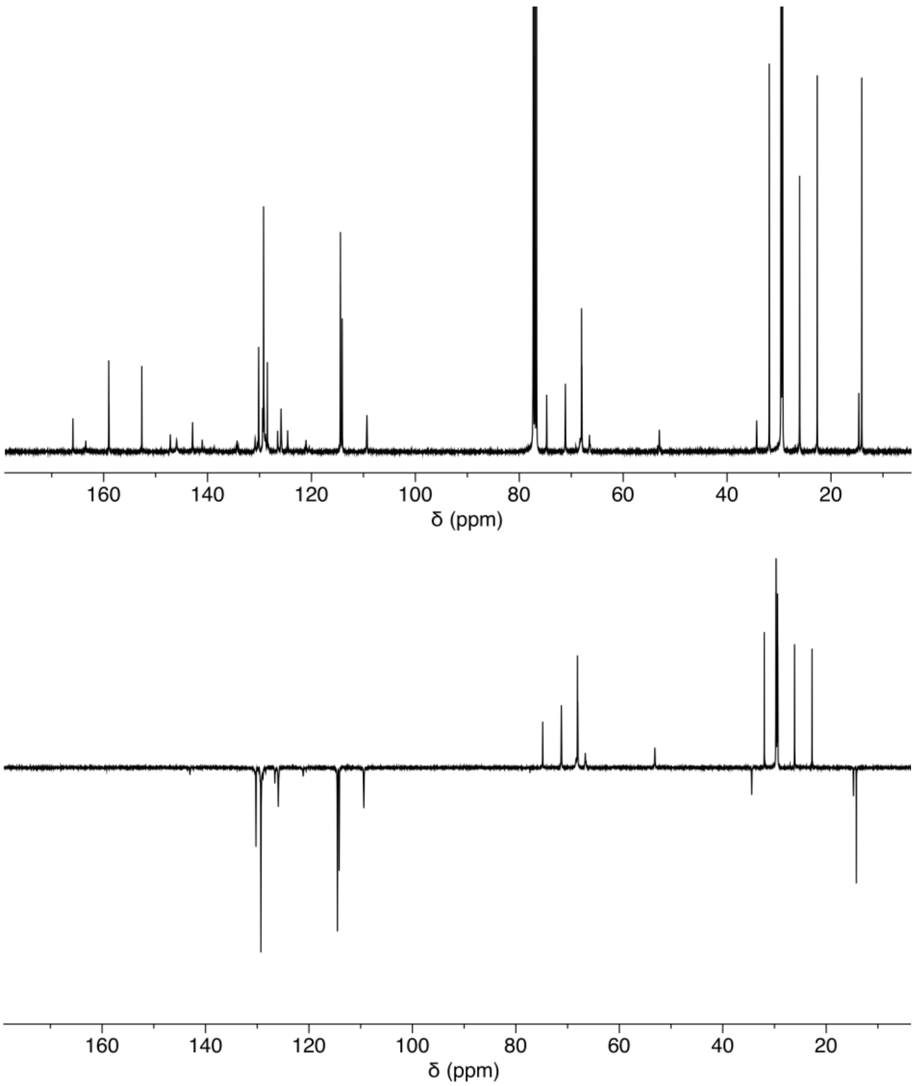

Fig. S8 ^{13}C NMR (top) and DEPT (bottom) spectra of compound **5d** ($CDCl_3$, 100 MHz).

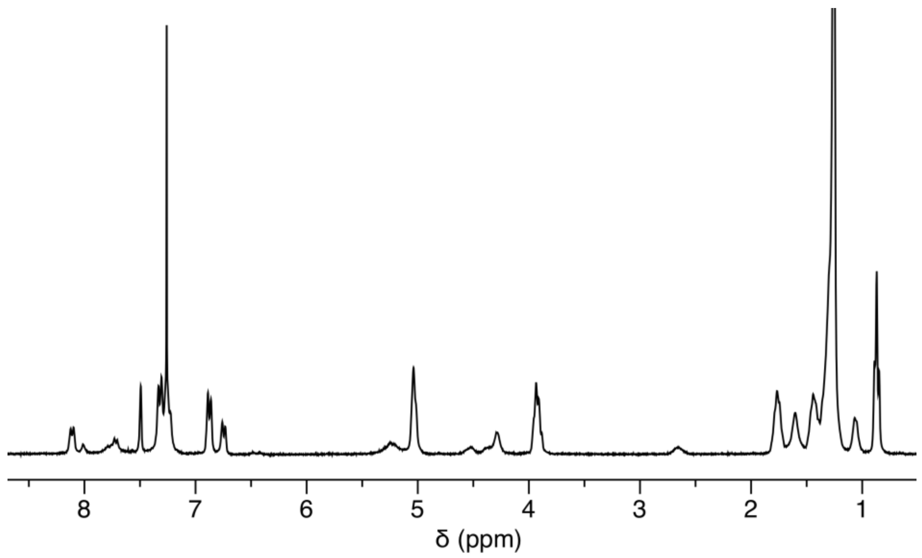

Fig. S9 ^{1}H NMR spectrum of compound **5e** ($CDCl_3$, 400 MHz).

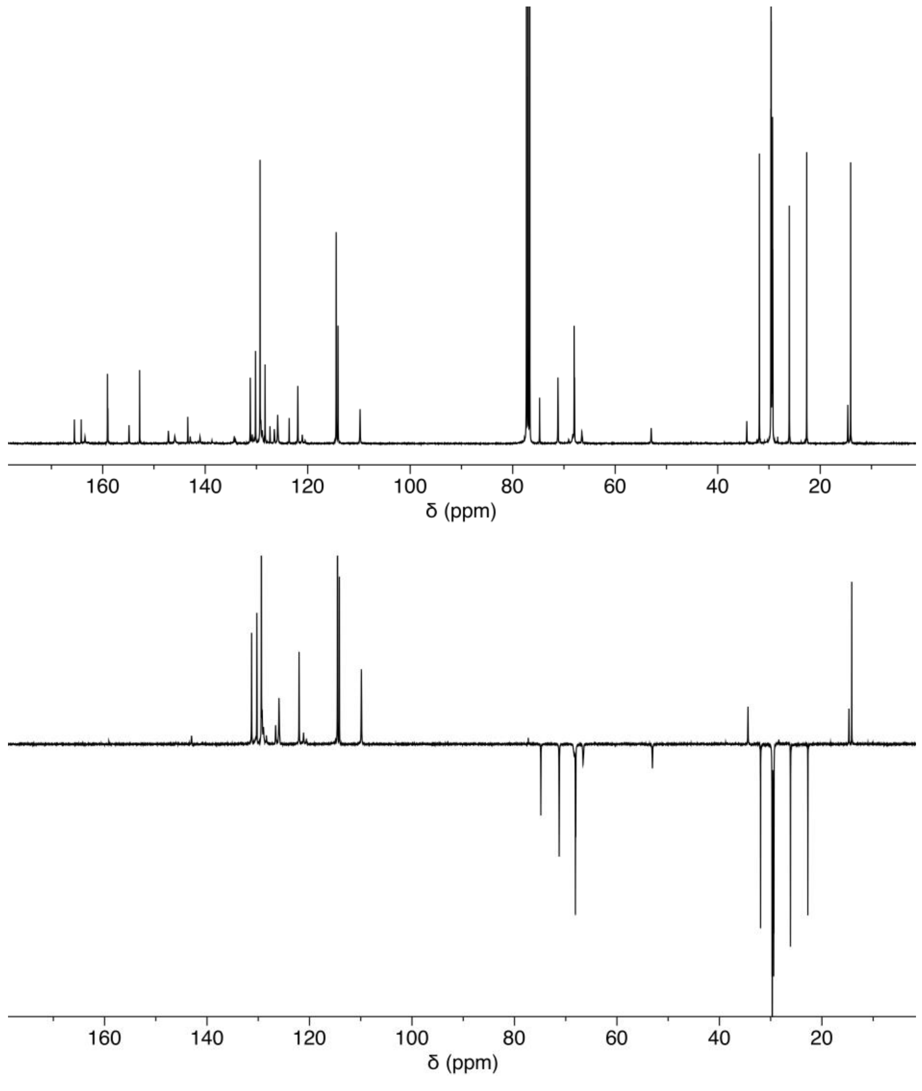

Fig. S10 ^{13}C NMR (top) and DEPT (bottom) spectra of compound **5e** ($CDCl_3$, 100 MHz).

3. UV-vis Spectra of Compounds 5a–e

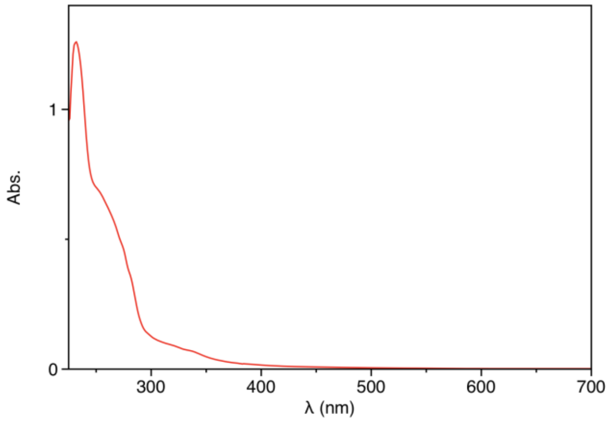

Fig. S11 UV-vis spectrum of compound **5a** (CH_2Cl_2).

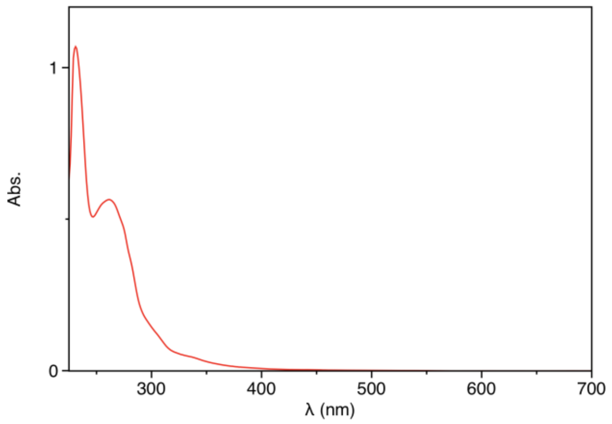

Fig. S12 UV-vis spectrum of compound **5b** (CH_2Cl_2).

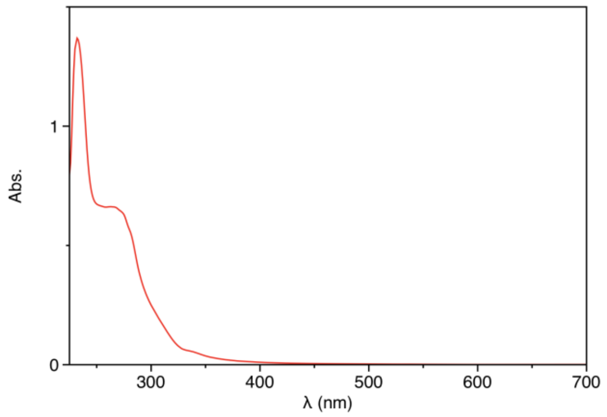

Fig. S13 UV-vis spectrum of compound **5c** (CH_2Cl_2).

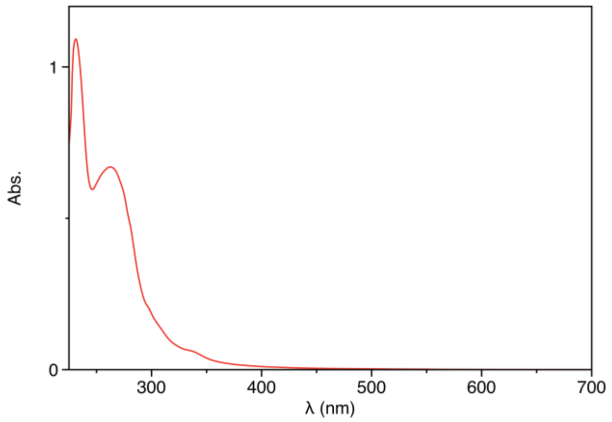

Fig. S14 UV-vis spectrum of compound **5d** (CH_2Cl_2).

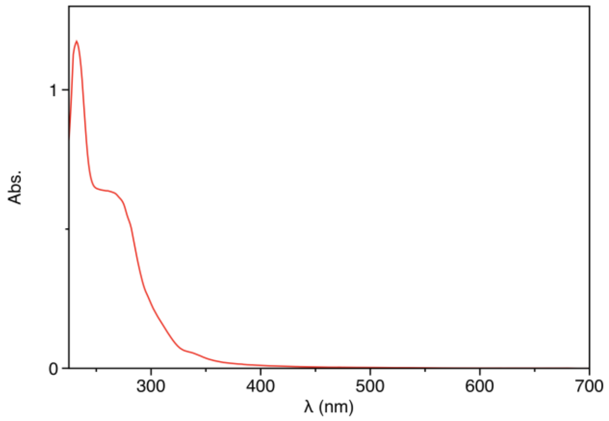

Fig. S15 UV-vis spectrum of compound **5e** (CH_2Cl_2).

4. MALDI-TOF Spectra for Compounds 5b and 5c

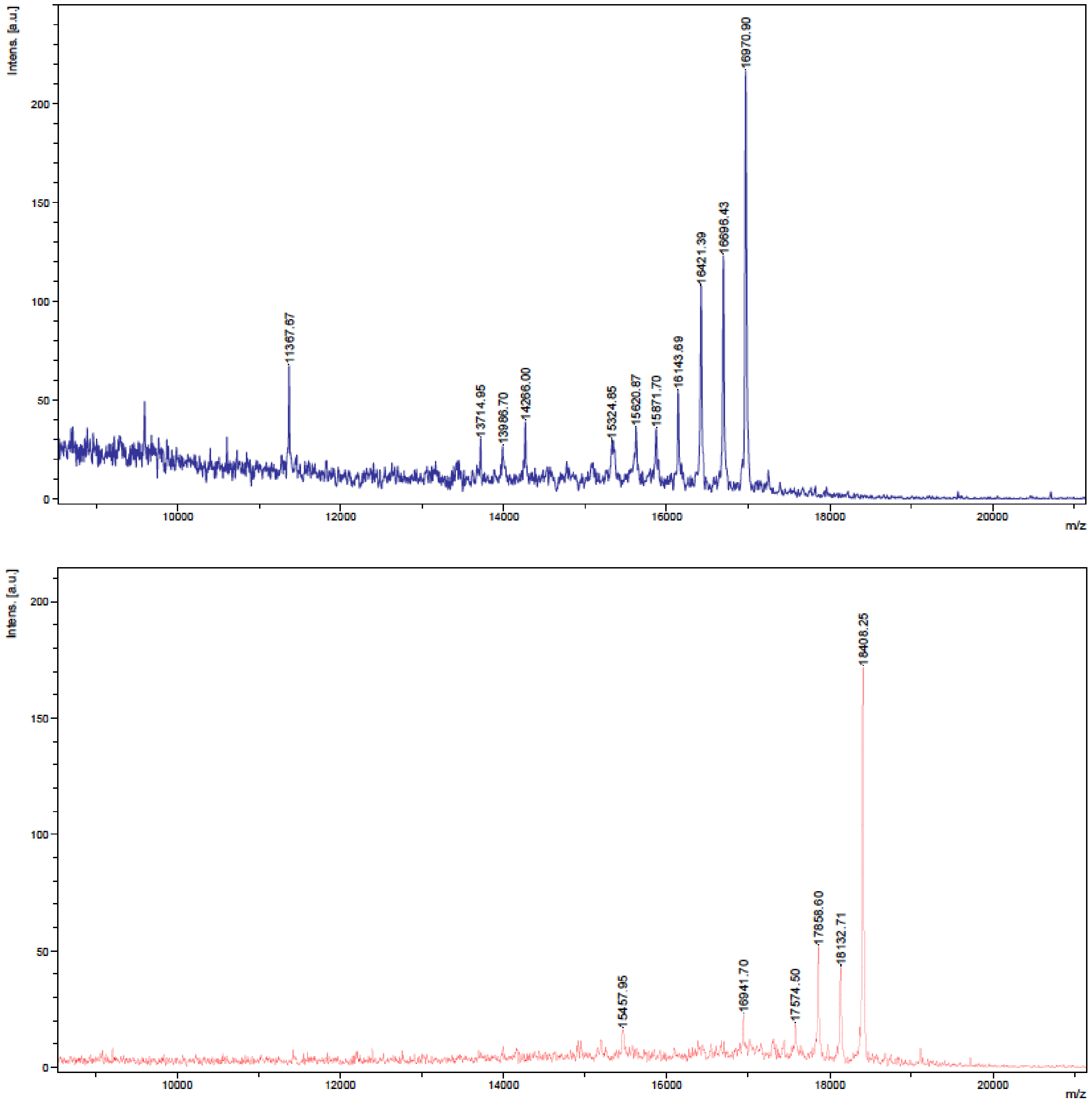

Fig. S16 MALDI-TOF mass spectra of compounds **5b** (top) and **5c** (bottom) showing the expected molecular ion peaks as well as fragments resulting from the successive loss of dodecyloxybenzyl subunits ($[M - (C_{12}H_{25}OPhCH_2)_n]^+$, with n = 1 to 4).

Under the same experimental conditions as those employed to obtain the MALDI-TOF spectra in Fig. S16, the molecular ion peak could not be detected for the molecules incorporating the shortest spacer (**5a**, **5d** and **5e**). This is not the result of high levels of fragmentation, as characteristic fragments were also not observed, but may be related to aggregation effects preventing the transfer of the compounds or fragments thereof in the gas phase during MALDI-TOF analysis.

5. Optical Microscopy on Compounds 5a–c

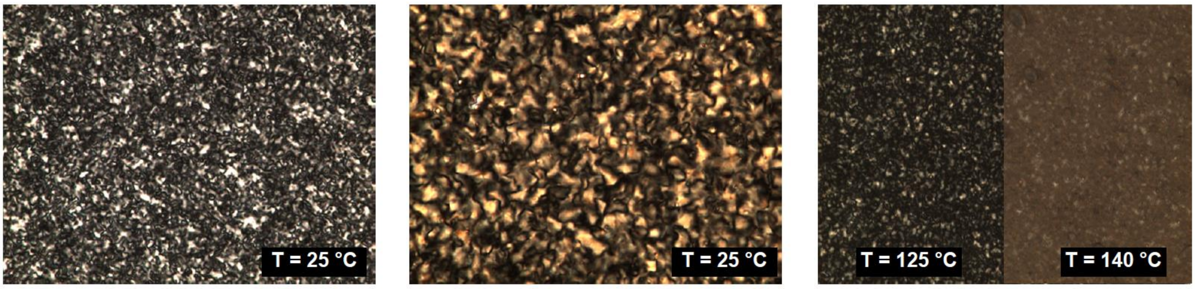

Fig. S17 Thermal optical micrographs collected at the indicated temperatures after cooling at 1 °C/min. from the isotropic liquid (right: **5a**, centre: **5b**, right: **5c**).

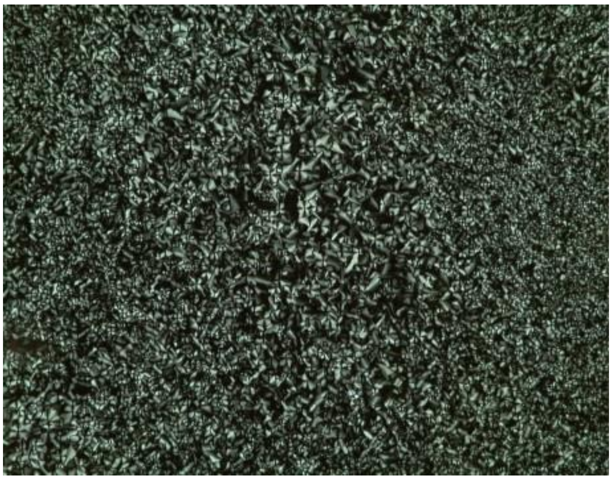

Fig. S18 Thermal optical micrograph of the pseudo focal conic fan texture displayed by **5b** in the hexagonal columnar phase upon cooling (5 °C/min) from the isotropic liquid to 108 °C.

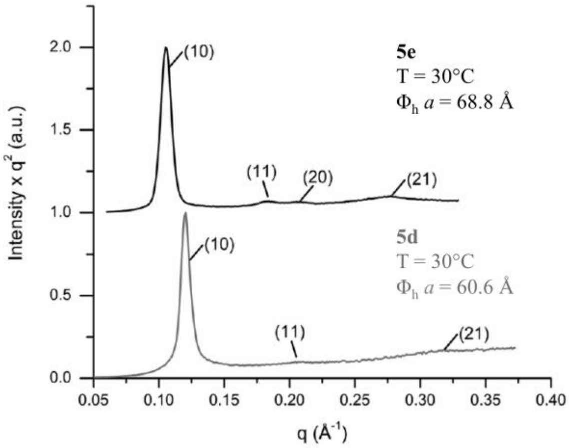

Fig. S19 Small-angle X-ray powder diffraction plots of **5d-e** collected in the columnar hexagonal phases. Compounds, collection temperature, diffraction peaks and lattice dimension are indicated.

6. XRD Data for Compounds 5a–e

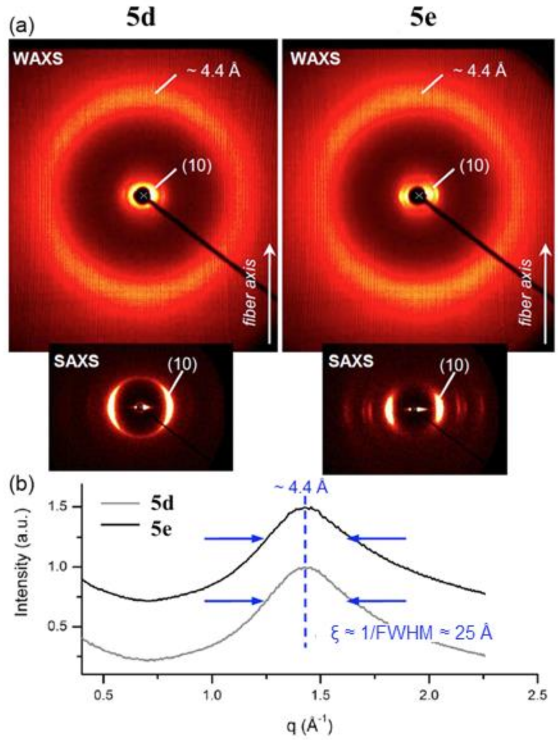

Fig. S20 Wide- and small-angle X-ray diffraction patterns collected from oriented fibers of the dendronized fullerenes with chiral spacers in the columnar hexagonal phases collected at 25 °C (a) and corresponding meridional plots integrating the diffuse wide-angle features observed at 4.4 Å (b). In (b) the average correlation length of the 4.4 Å diffuse features marked in (a), calculated from the full width of the half maxima, indicate that the chain-chain correlation length is about 25 Å.

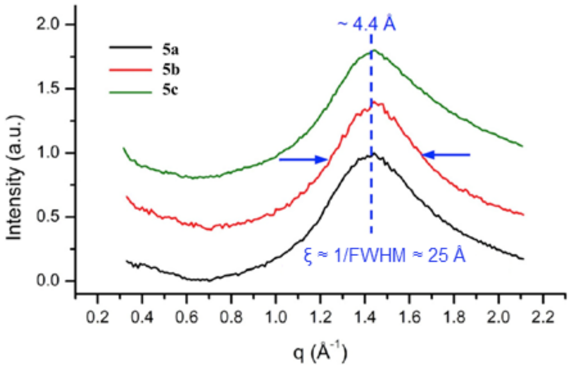

Fig. S21 Wide-angle diffraction plots integrating the wide-angle meridional region of the oriented fiber patterns shown in Fig. 2. The average correlation length of the 4.4 Å diffuse features marked in Fig. 2, calculated from the full width of the half maxima, indicate that the chain-chain correlation length is about 25 Å.

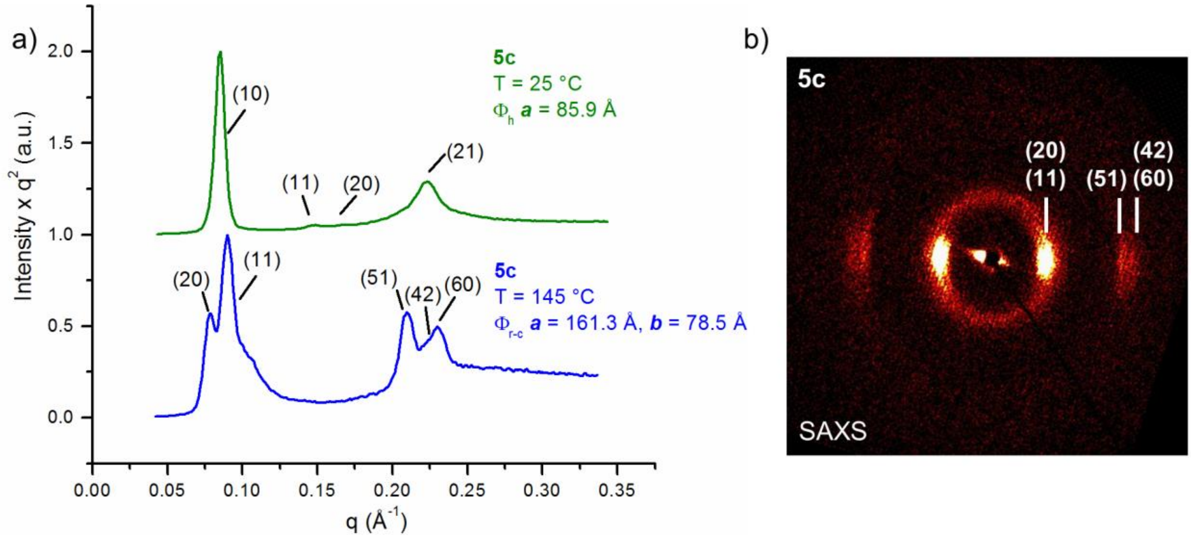

Fig. S22 Representative small-angle powder diffraction plots (a) and fibre diffraction plots (b) of **5c**. Powder diffraction plots (a) were collected in the hexagonal columnar phase at 25 °C (top) and rectangular columnar phase at 145 °C (bottom).

7. Molecular Modelling for Compounds 5b–d

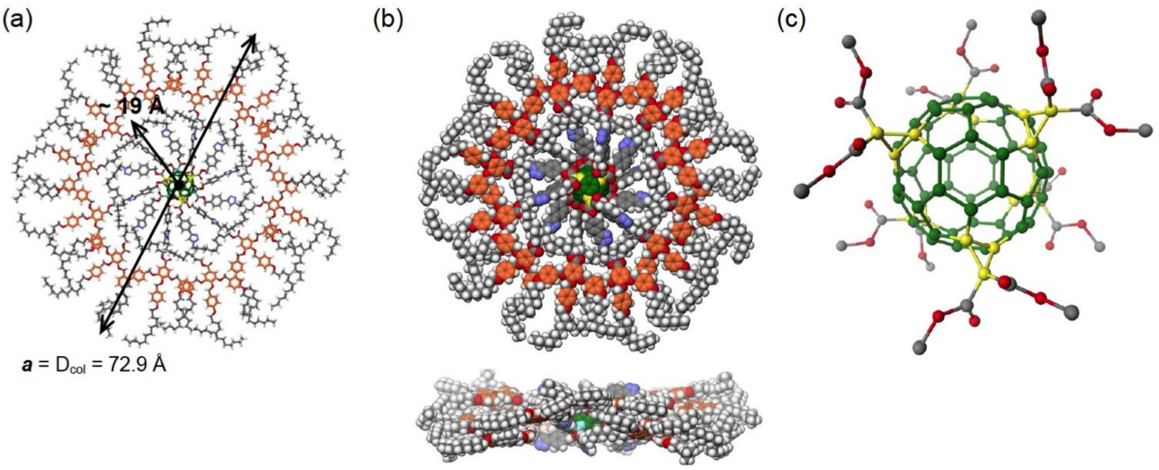

Fig. S23 Molecular model of the dendronized [60]fullerene **5b**: top view of the column strata (a), top and side views of the column strata shown in space filling (b), and detail view of the core region (c). Color code: Gray as C, white as H, red as O, and blue as N; Orange as dendron aromatic rings, green as fullerene core, and yellow as the sp^3 C atoms linkage C_{60}-dendrons.

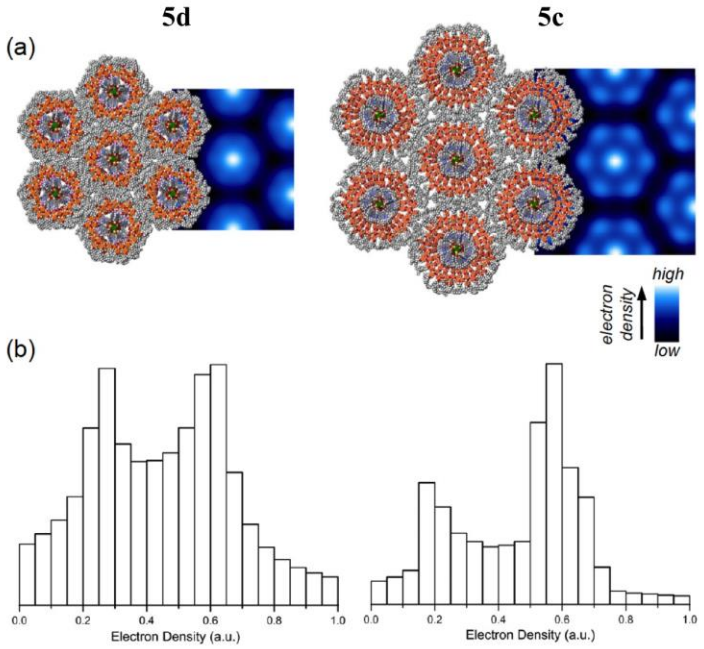

Fig. S24 Molecular models shown at scale with the reconstructed relative electron density maps (a) and the corresponding histograms of the electronic density distribution within the columnar hexagonal unit cell confirming the diffraction peaks phase assignment (10)+, (11)–, (20)–, and (21)+ (b).

8. Solution CD Data for Compound 5d

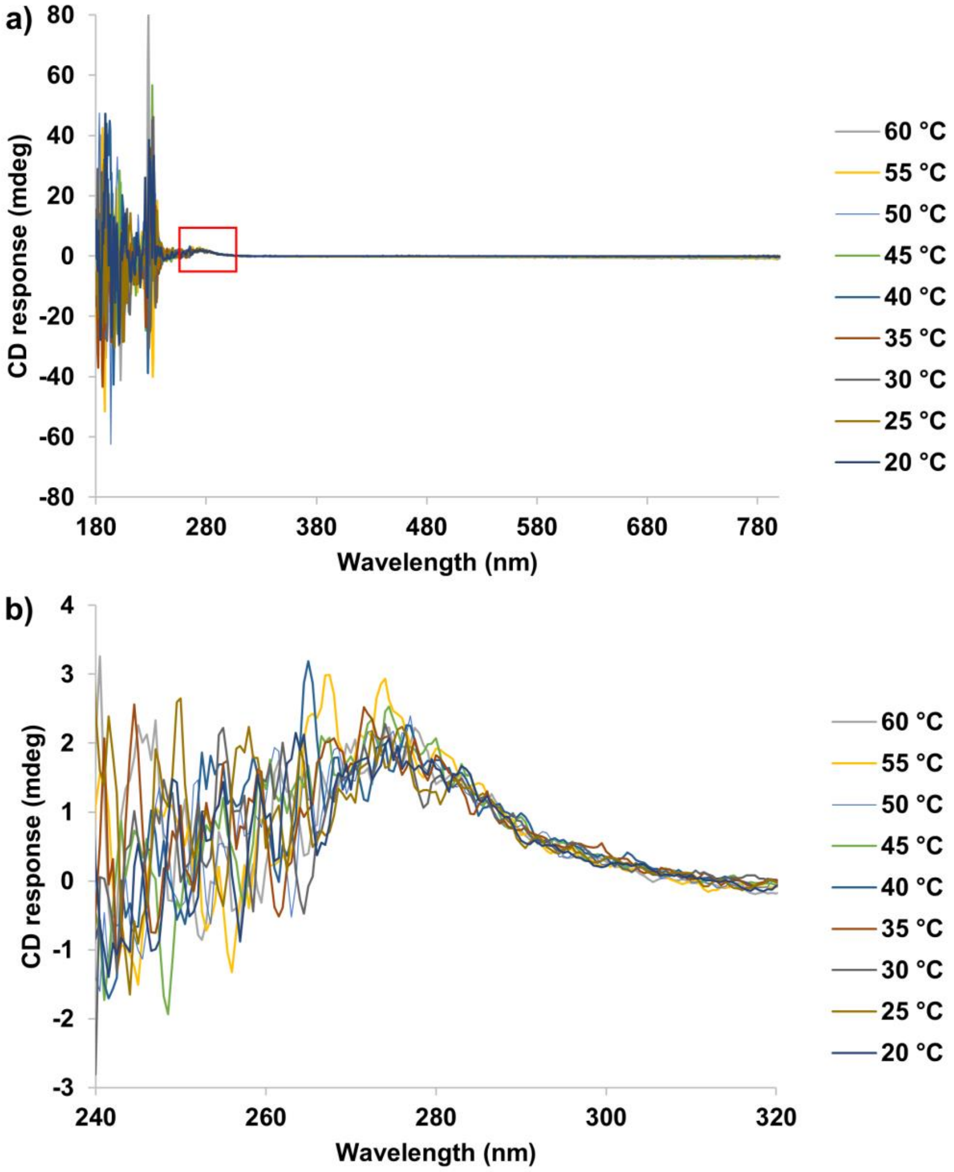

Fig. S25 Solution CD spectra of **5d** (6.0×10^{-5} M in *n*BuOH/MCH (7:3)) upon cooling from 60 to 20 °C at a rate of 0.5 °C/min (a). The red box in (a) indicates the region of the spectra expanded and shown in (b).

9. References

1. V. Percec, W.-D. Cho, G. Ungar, D. J. P. Yeardley, *J. Am. Chem. Soc*. 2001, **123**, 1302–1315.
2. B. Dardel, D. Guillon, B. Heinrich, R. Deschenaux, *J. Mater. Chem*. 2001, **11**, 2814–2831.
